# Taxonomic revision of *Bracalba* Dodd (Hymenoptera, Platygastridae s.l.), a parasitoid wasp genus endemic to Australia

**DOI:** 10.3897/zookeys.236.3434

**Published:** 2012-10-02

**Authors:** Roger A. Burks, Lubomír Masner, Norman F. Johnson, Andrew D. Austin

**Affiliations:** 1 Department of Evolution, Ecology, and Organismal Biology, The Ohio State University, 1315 Kinnear Road, Columbus, OH 43212, U.S.A.; 2 Agriculture and Agri-Food Canada, K.W. Neatby Building, Ottawa, ON K1A 0C6, Canada; 3 Australian Centre for Evolutionary Biology and Biodiversity, School of Earth and Environmental Sciences, The University of Adelaide, SA 5005, Australia

**Keywords:** Platygastroidea, Platygastridae s.l., Scelionidae, *Bracalba*, Scelioninae, key, revision, database, endemism, parasitoid

## Abstract

The endemic Australian parasitic wasp genus *Bracalba* (Hymenoptera: Platygastridae) is revised. Sixteen species are recognized: *Bracalba cuneata* Dodd, *Bracalba laminata* Dodd and *Bracalba nigrescens* (Dodd) are redescribed and thirteen new species are recognized; *Bracalba clavata* Burks, **sp. n.**, *Bracalba globosa* Burks, **sp. n.**, *Bracalba hesperia* Burks, **sp. n.**, *Bracalba intermedia* Burks, **sp. n.**, *Bracalba magnirubra* Burks, **sp. n.**, *Bracalba parvirubra* Burks, **sp. n.**, *Bracalba pinnula* Burks, **sp. n.**, *Bracalba plana* Burks, **sp. n.**, *Bracalba propodealis* Burks, **sp. n.**, *Bracalba sculptifrons* Burks, **sp. n.**, *Bracalba sparsa* Burks, **sp. n.**, *Bracalba tricorata* Burks, **sp. n.**, and *Bracalba tridentata* Burks, **sp. n.** The genus is found continent-wide but mostly south of the Tropic of Capricorn, and with the highest species diversity occurring in the Pilbara and south-western regions of Western Australia. The hosts of *Bracalba* are unknown but specimens reared from eggs confirm that the genus is associated with orthopteran hosts. A preliminary phylogeny of species did not indicate that species groups were monophyletic, but they are retained despite paraphyly because they are convenient for specimen identification.

## Introduction

The Platygastroidea are a highly diverse component of the Australian parasitoid wasp fauna with 666 described species in 80 genera (ABRS 2012). However, like most parasitoid families, the real species richness of the group is very much larger than this. The scope of this disparity was recently indicated in a revision of *Idris (Ceratobaeus)* (Iqbal and Austin 2000) that resulted in an initial four-fold increase in species to 130+ taxa, and this increase appears to generally hold true for revisions of other platygastroid genera.

The first Australian scelionids were described by Walker (1839) from material collected by Charles Darwin along the east coast of the continent. However, the majority of Australian species (63%) were described by A.P. Dodd between 1913 and 1939 and, testament to his taxonomic abilities, virtually all of his species remain valid, although many have been transferred to other genera (Johnson 1992; ABRS 2012). Other than the taxonomic treatment of a number of small genera by various authors, the most influential post-Dodd studies on the Australian fauna have been the revision of the telenomine genera *Psix* (Johnson and Masner 1985) and *Trissolcus* (Johnson 1991), the spider-associated genera *Baeus* (Stevens and Austin 2007), *Idris (Ceratobaeus)* (Iqbal and Austin 2000) and *Mirobaeoides* (Austin 1986), the acridid egg parasitoid genus *Scelio* (Dangerfield et al. 2001), and the generic overview provided by Galloway and Austin (1984). Platygastroids are mostly endoparasitoids of insect and spider eggs, including all taxa previously accommodated in the family Scelionidae, while many platygastrids (*s.str*.) oviposit either in the egg or early larva of gall midges (Cecidomyiidae) and complete their development in the larvae (Austin and Field 1997; Austin et al. 2005).

The current study focuses on one of the few genera, *Bracalba* Dodd, that is endemic to the Australian continent. Among other characters, the genus is recognised by its stout, sculptured body, obviously setose eyes, face with pronounced frontal depression, and very long fore wing postmarginal vein (R1). Previously known from only three species, two from south-eastern Queensland and one from south-western Western Australia, this revision treats 16 species in total. In addition, we document the distribution of all species, present a key to their identification, and undertake a preliminary analysis of species-level relationships.

The contributions of the individual authors are as follows; R.A. Burks: character definition, species concept development; key development, imaging, capture of specimen data, manuscript preparation, phylogenetic analysis and illustration; L. Masner: specimen acquisition, and generic overview; N.F. Johnson: generic concept development and manuscript preparation ; A.D. Austin: initial species concept development, manuscript preparation, and taxonomic overview.

## Materials and methods

Specimens examined were provided by the following collections: The American Entomological Institute, Gainesville, Florida, USA (AEIC)^1^; Australian Museum, Sydney, Australia (AMSA)^2^; Australian National Insect Collection, Canberra, Australia (ANIC)^3^; The Natural History Museum, London, United Kingdom (BMNH)^4^; Canadian National Collection of Insects, Arachnids and Nematodes, Ottawa, Canada (CNCI)^5^; Museum of Victoria, Entomology, Melbourne, Australia (MVMA)^6^; Museum of Comparative Zoology, Harvard University, Cambridge, Massachusetts, USA (MCZC)^7^; C.A. Triplehorn Insect Collection, Ohio State University, Columbus, Ohio (OSUC)^8^; Queensland Primary Industries and Fisheries Insect Collection, Indooroopilly, Australia (QDPC)^9^; Queensland Museum, Brisbane, Australia (QMBA)^10^; R.M. Bohart Museum, University of California, Davis, USA (UCDC)^12^; Western Australian Museum, Perth, Australia (WAMP)^13^; Waite Insect and Nematode Collection, Adelaide, Australia (WINC)^14^. South Australian and Northern Territory holotypes for species newly described in this manuscript are deposited in the South Australian Museum, Adelaide, Australia (SAMA)^11^.

This revision is a product of the Platygastroidea Planetary Biodiversity Inventory, funded by the U.S. National Science Foundation (N.F. Johnson, Ohio State University; A.D. Austin, University of Adelaide; Principal Investigators). One objective of this project is to use biodiversity informatics resources to accelerate taxonomic processes, making real-time collaboration possible within a community of researchers. Data associated with specimens examined in this study can be accessed at hol.osu.edu and by entering the unique specimen identifier (e.g. OSUC 238448) in the search form. Morphological terminology follows Mikó et al. (2007), except for: 1) antennal features discussed by Bin (1981), 2) anteclypeus and postclypeus used sensu Dangerfield et al. (2001), 3) dorsal epomial carina and vertical epomial carina, which are mentioned by Masner and Johnson (2007) and defined by Talamas et al. (2011), 4) a newly coined “metasomal bend,” which is a ventrad bend occurring near the midlength of the 4th metasomal segment, and 5) ovipositor features described by Austin and Field (1997). Surface sculptural terms are used in part based on Eady (1968), with general terms added for descriptive purposes: “mesh” to define a polygonal, ovate or circular area enclosed by raised or sunken borders; “septa” to define distinctive borders that enclose meshes; and “interspaces” to define areas separating individual sculptural features. Note that sculptural septa can be differentiated, as in the longitudinal septa being much stronger than the transverse septa. They are also repeated corresponding to the number of sculptural meshes present. The aforementioned terminology is used instead of calling all raised sculpture “carinae,” which would remove all recognizable meaning from the word. Instead, “carina” is used for a raised sculptural element that does not correspond to repeated enclosed meshes. Life science identifiers (LSIDs) can be resolved at http://lsid.tdwg.org (i.e. urn:lsid:zoobank.org:act:E5095F58-4603-4796-9D22-03A759B7B29).

**Illustration and data citations.** Photographs were taken using a Visionary Digital BK+ Imaging System, November 2010 model, with either a K2 Long Distance Microscope or a 65 mm varifocal lens. Source photos were stacked using Zerene Stacker version 1.04 and enhanced using Adobe Photoshop CS5.

**Phylogenetic analysis.** An exact search using implicit enumeration (branch-and-bound) was performed using TNT (Tree analysis using New Technology) version 1.1 (Goloboff et al. 2003, 2008). Bootstrapping was performed with 1,000 replicates using New Technology searches at an initial level of 95. The outgroup specimens coded in this analysis were from an undescribed species of *Chromoteleia* Ashmead from Belize, (OSUC 064028, OSUC 064021), chosen because of the overall morphological similarity between *Chromoteleia* and *Bracalba*. A total of 42 characters were used, of which 38 were parsimony informative (see Appendix II for characters and matrix).

## Taxonomy

### Bracalba

Dodd

urn:lsid:zoobank.org:act:E5095F58-4603-4796-9D22-03A759B7B29

urn:lsid:biosci.ohio-state.edu:osuc_concepts:457

http://species-id.net/wiki/Bracalba

Bracalba Dodd 1931: 78 (original description. Type: *Bracalba laminata* Dodd, by original designation); Muesebeck and Walkley 1956: 336 (citation of type species); Masner 1976: 22, 23 (description, key to separate *Baryconus* Förster, *Bracalba* Dodd, *Chromoteleia* Ashmead, *Oxyscelio* Kieffer); Galloway and Austin 1984: 8, 13 (diagnosis, list of species described from Australia, keyed); Johnson 1992: 354 (catalog of world species); Austin and Field 1997: 18, 68 (structure of ovipositor system, discussion of phylogenetic relationships, genus misplaced in Baryconini).

#### Diagnosis.

Eye setose; frontal depression present; antenna 12-segmented; netrion present; postmarginal vein of fore wing present, longer than marginal and stigmal veins; mesotibia and metatibia each with 1 spur; metascutellum setose dorsally and ventrally; ovipositor *Scelio*-type.

#### Description.

Body length: 2.75–6.88 mm (n=78).

**Head**. Head shape in dorsal view: transverse. Hyperoccipital carina: absent. Occipital carina: present laterally, broadly interrupted medially; present, complete medially. Occipital carina sculpture: crenulate. OOL: lateral ocellus nearly contiguous with inner orbits, OOL < 0.5 OD. Upper frons: convex, without frontal shelf. Scrobe shape: frons with shallow unmargined depression above antennal foramina. Frons sculpture: areolate rugose, transversely striate within scrobe; areolate rugose, scrobe sparsely punctate. Submedian carina: absent. Orbital carina: absent. Inner orbits: diverging ventrally. IOS/EH: IOS slightly less than EH. Interantennal process: triangular in lateral view, well-developed. Central keel: absent. Antennal foramen opening: nearly anteriorly. Lower frons striae: absent. Malar sulcus: present. Compound eye size: of normal proportions, not significantly reduced. Compound eye setation: densely setose; sparsely setose. Gena: narrow, weakly convex, receding behind posterior orbit. Clypeus shape: transversely rectangular. Apical margin of clypeus: rounded; with a small median point. Anteclypeus: absent. Postclypeus: absent. Labrum: not visible. Mandible shape: moderate. Mandibular teeth: apex with 2, acute, subequal teeth; apex tridentate, teeth acute, middle tooth distinctly shortest. Arrangement of mandibular teeth: transverse. Number of maxillary palpomeres: 4. Shape of maxillary palpomeres: cylindrical. Number of labial palpomeres: 2.

**Antenna**. Number of antennomeres in female: 12. Number of antennomeres in male: 12. Insertion of radicle into A1: parallel to longitudinal axis of A1. Shape of A1: more or less cylindrical, not flattened. Length of A3 of female: distinctly longer than A2. Number of clavomeres in female antenna: 8. Claval formula of female antenna: A12-A7/1-2-2-2-2-2; A12-A6/1-2-2-2-2-2-2; A12-A6/1-2-2-2-2-2-1. Arrangement of doubled multiporous plate sensilla on female clava: in longitudinal pairs. Tyloid distribution on male antenna: A5 only. Shape of male flagellum: filiform.

**Mesosoma**. Mesosoma shape in dorsal view: longer than wide. Mesosoma shape in lateral view: longer than high. Medial portion of transverse pronotal carina: absent. Posterior apex of pronotum in dorsal view: straight, bifid apically to articulate with tegula. Vertical epomial carina: absent. Dorsal epomial carina (lateral portion of transverse pronotal carina of Vilhelmsen et al. (2010)): absent. Central pronotal area: vertical, not visible in dorsal view. Lateral face of pronotum: weakly concave below position of dorsal epomial carina. Netrion: present. Netrion shape: moderately wide, closed ventrally. Anterior portion of mesoscutum: vertical, flexed ventrally to meet pronotum. Mesoscutum shape: pentagonal in outline, posterolateral corner rounded. Skaphion: absent. Notauli: present, percurrent. Parapsidal lines: absent. Admedial lines: absent. Transscutal articulation: well-developed, crenulate. Shape of mesoscutellum: quadrate to trapezoidal. Armature of mesoscutellum: absent. Surface of mesoscutellum: convex throughout. Median longitudinal furrow on mesoscutellum: absent; present. Shape of axillula: small, dorsal margin sinuate. Metascutellum in dorsal view: clearly differentiated. Metascutellar armature: produced into flattened plate. Metascutellar setation: setose dorsally and ventrally. Metapostnotum: not defined externally. Extent of metasomal depression of propodeum: percurrent, extending anteriorly to anterior margin of propodeum. Lateral propodeal projection: well-developed, extending clearly beyond anterior margin of T1. Mesopleural carina: absent or strongly abbreviated, present only near mid coxa. Mesal course of acetabular carina: projecting anteriorly, but too short to intercede between fore coxae. Mesopleural pit: absent. Sternaulus: absent. Posterodorsal corner of mesopleuron: rounded anteriorly.

**Legs**. Number of mid tibial spurs: 1. Number of hind tibial spurs: 1. Dorsal surface of hind coxa: smooth. Hind tibia shape: cylindrical, ecarinate. Trochantellus: indicated by transverse sulcus on femur.

**Wings**. Wing development of female: macropterous. Wing development of male: macropterous. Tubular veins in fore wing: present. Bulla of fore wing R: absent. Extent of marginal venation of fore wing: distinct marginal or postmarginal veins developed. Origin of r-rs in fore wing: arising at junction of R/R1 with costal margin. Development of basal vein (Rs+M) in fore wing: nebulous, weakly pigmented. Development of R in hind wing: elongate, extending to costal margin.

**Metasoma**. Number of external terga in female: 6. Number of external sterna in female: 6. Number of external terga in male: 8. Number of external sterna in male: 7. Shape of metasoma: acuminate, widest submedially. Laterotergites: present, narrow. Laterosternites: present. T1 of female: raised medially into low, rectangular or subelliptical platform, laterally depressed. Relative size of metasomal terga: T2–T4 largest, subequal in size. Terga with basal crenulae: T1–T4. Sublateral carinae on terga: absent. Median longitudinal carina on metasomal terga: absent; present. Distribution of felt fields: absent. Ovipositor type: Scelio-type (Austin and Field 1997).

#### Etymology.

Dodd did not specify the source of the name for this genus, but presumably it is derived the name of the Queensland town of Bracalba. (Dodd also used the names of Merriwa, NSW and Nyleta, QLD for other genera.) He originally combined this name with two species epithets of variable gender, both coined in feminine form: *Bracalba cuneata* and *Bracalba laminata*, clearly indicating that he intended *Bracalba* to be a feminine noun.

#### Distribution.

**Figure 1.**
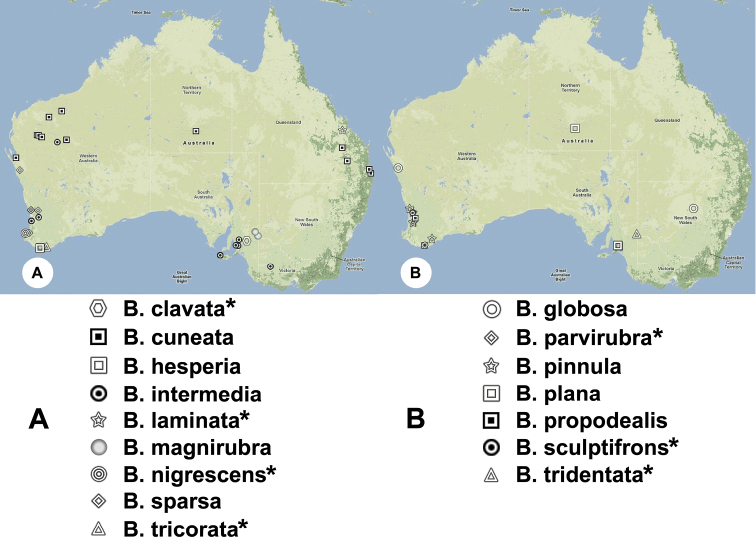
Collection events for each *Bracalba* species. * = species known from only one locality.

*Bracalba* has been collected only from Australia (Fig. 1), and has seldom been collected north of the Tropic of Capricorn. The furthest north that any *Bracalba* has been collected was at 24°39'S, and only two species have been found north of Alice Springs, NT. Location records show that the highest species diversity occurs in the Pilbara and south-western regions of Western Australia.

#### Biology.

The hosts of *Bracalba* are unknown but the structure of the ovipositor (*Scelio*-type; Austin and Field 1997) and specimens reared from eggs confirm that the genus is associated with orthopteran hosts. These eggs could not be identified beyond the ordinal level.

#### Relationships among species.

**Figure 2.**
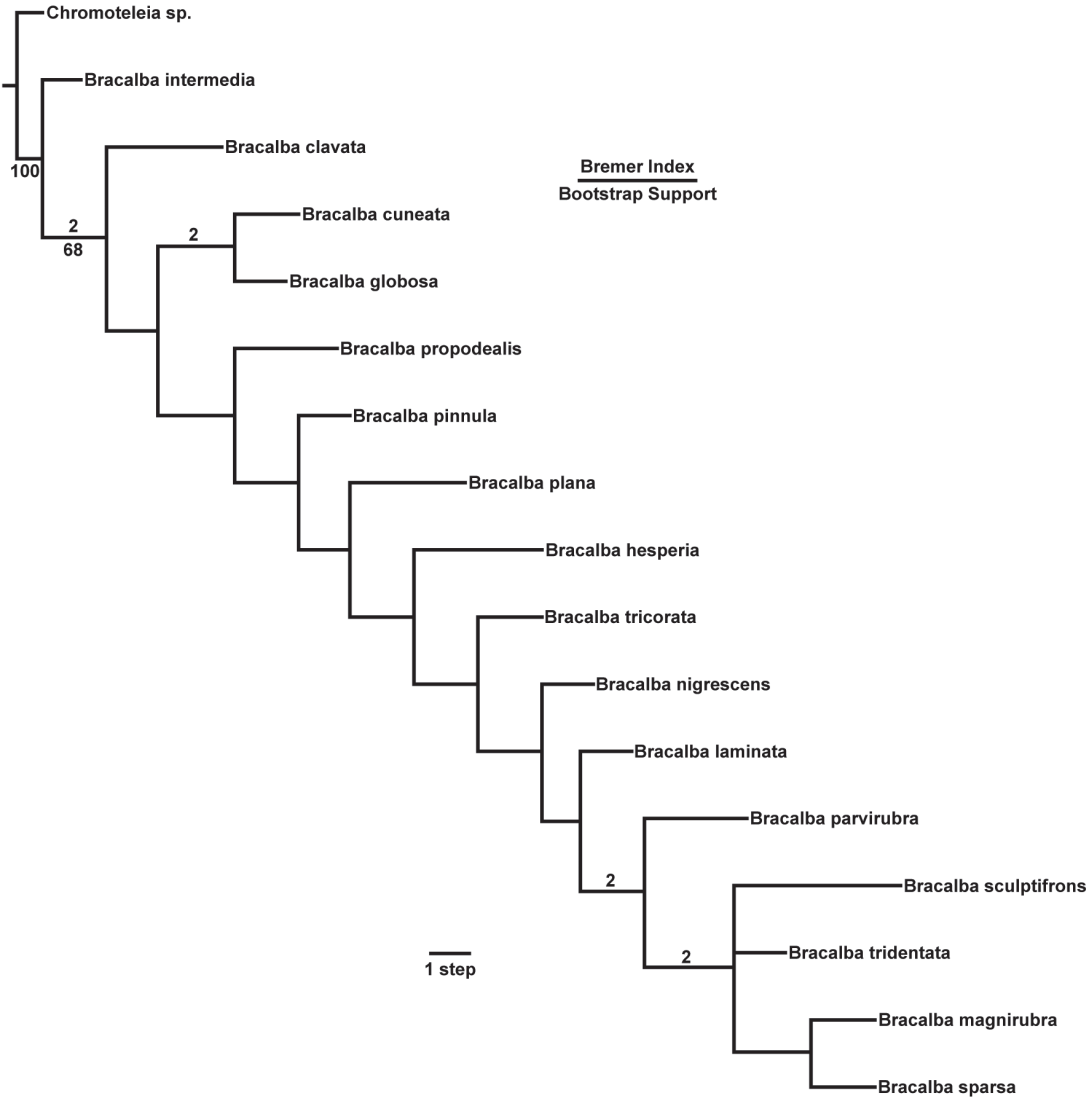
Strict consensus phylogram of two most parsimonious trees for species of *Bracalba* using implicit enumeration (branch-and-bound exact search), score = 166. Bootstrap support values found using TNT new technology search (set initial level = 95). Bremer support values above 1, and bootstrap values about 50% indicated on branches.

The implicit enumeration search found two optimum trees of 166 steps (strict consensus: Fig. 2). *Bracalba* was monophyletic with respect to the *Chromoteleia* outgroup (bootstrap = 100). Intuitively-based species groups were not monophyletic in the analysis, but this was likely due to homoplasy resulting from inclusion of many sculptural and coloration characters that have mainly descriptive value. Species with a bend at metasomal segment 4 in females did not form a monophyletic group, but this was complicated by the smooth transition in this character from being distinctively present to clearly absent. One feature that was helpful in determining species group, but which was not reported in the key because it was too difficult to accurately and consistently assess, was the lateral margin of the dorsal axillar area. In the *cuneata*-group, the dorsal axillar area was essentially triangular, broadening posteriorly, with the lateral margin sometimes forming a posterior tooth. In the *laminata*-group the dorsal axillar area was generally more nearly semicircular, broadest at midlength, and with the lateral margin not forming a posterior tooth. Apparent variation, and difficultly in observing this character with confidence, caused us to omit it from the key.

#### Key to species of *Bracalba*

**Note:** It is imperative to consult figures when using the below key, as shape features of the metascutellum and metasoma are very important in identifying *Bracalba* species with any hope of accuracy.

**Table:** 

1	Mandibles bidentate with no hint of a third tooth (Fig. 11), teeth narrowly separated and of nearly the same length. Females: T4 without bend or hump (Figs 4, 7); fore wings extending to middle of T5 or beyond when folded over body	2 (*cuneata*-group)
–	Mandibles either tridentate with a small middle tooth (Fig. 40) or very rarely bidentate, but with teeth broadly separated and/or dorsal tooth much longer than ventral tooth (Figs 52, 58). Females: T4 with or without bend or hump (bend: Fig. 33, hump: Fig. 56); fore wings extending only to middle or apex of T4 when folded over body	4 (*laminata*-group)
2	Metascutellum flat (Figs 12–13), not projecting dorsally. Male: A3 short: 1.1–1.2× as long as broad (Fig. 13)	*Bracalba plana* Burks, sp. n.
–	Metascutellum projecting dorsally (Figs 4, 7). Male: A3 over 1.5× as long as broad	3
3	Mesoscutellum sparsely foveolate, with smooth interspaces (Fig. 8). Mesoscutum smoothly and regularly convex anteriorly and posteriorly (Figs 7–8)	*Bracalba globosa* Burks,sp. n.
–	Mesoscutellum densely foveolate, interspaces with irregular longitudinal rugae (Fig. 3). Mesoscutum abruptly convex only anteriorly, flattened or irregularly convex posteriorly (Figs 3–4)	*Bracalba cuneata* Dodd
4	Propodeum with sharp protrusions (from lateral propodeal area) protruding dorsally immediately posterior to metascutellum, reaching at least the same height as the dorsal surface of metascutellum (Figs 47–48). Males unknown	*Bracalba propodealis* Burks,sp. n.
–	Propodeum without such protrusions	5
5	Metascutellum strongly narrowed apically (Figs 28, 32, 36, 40, 55), tapering to an incised or narrowly truncate apex that is narrower than the maximum metascutellar length; metasomal bend or hump present in females (Figs 33, 56)	6
–	Metascutellum with apex broadly truncate, as broad or broader than total metascutellar length (Figs 14, 19, 24, 43, 47, 60); metasomal bend present or absent in females	10
6	Mesosoma truncate anteriorly (Figs 28, 32), with antero-lateral pronotal corners protruding anteriorly. Metascutellum incised apically	7
–	Mesosoma smoothly rounded anteriorly (Fig. 36), with antero-lateral pronotal corners much weaker, more rounded, and not protruding anteriorly. Metascutellar apex variable	8
7	Mesoscutellum without median carina (Fig. 32). Females with a mostly reddish metasoma and very large body (at least 6.5 mm long)	*Bracalba magnirubra* Burks,sp. n.
–	Mesoscutellum with median carina (Fig. 28). Color of metasoma in females unknown. Female body length 4.25 mm [using Dodd’s (1930) description, which is probably an underestimate]	*Bracalba laminata* Dodd
8	Antennal scrobes crossed by transverse carinae that meet at torular triangle (Fig. 38). Females unknown	*Bracalba nigrescens* (Dodd)
–	Antennal scrobes foveolate or smooth. Females with hump near midlength T4 (Figs 41, 56)	9
9	Antennal scrobes smooth and distinct. Clypeus with blunt median carina (Fig. 42). Female: S6 without apical incision (Fig. 42); T5, S5 with many strong longitudinal carinae (Figs 40, 42)	*Bracalba parvirubra* Burks,sp. n.
–	Antennal scrobes foveolate and indistinct (Fig. 57). Clypeus without median carina (Fig. 58). Female: S6 with strong apical incision (Fig. 59); T5, S5 with at most a median carina (Figs 55, 59)	*Bracalba sparsa* Burks,sp. n.
10	Frontal depression with strong longitudinal carina that is crossed by a strong transverse carina dorsally. Clypeus with median carina and transverse dorsal carina (Fig. 53). Metascutellum broadly trapezoidal (Fig. 51). Females: metasoma reddish, T5 and S5 with many longitudinal carinae, S6 without apical notch (Fig. 54)	*Bracalba sculptifrons* Burks, sp. n.
–	Frontal depression without such a set of carinae. Clypeus with weaker or no carinae (Figs 20, 45, 64). Metascutellum variable (Figs 19, 62). Females: metasoma brown to black, T5, S5, and S6 variable	11
11	Metascutellum elongate-trapezoidal with concave lateral margins (Fig. 62). Female: S6 with strong apical notch (Fig. 65); T5, S5 with distinct longitudinal carinae (Figs 62, 65). Males unknown	*Bracalba tridentata* Burks, sp. n.
–	Metascutellum broadly trapezoidal with straight lateral margins (Figs 19, 43, 60), or subrectangular to weakly convex with irregular lateral margins (Figs 14, 24). Female: S6 without apical notch (Figs 17, 23, 46); T5, S5 variable	12
12	Mesoscutellum with a set of incomplete median longitudinal grooves (Fig. 19). Middle mandibular tooth almost as long as the other two (Fig. 20). Clypeus with weak median carina. Female: S5 with longitudinal carinae (Fig. 23). Males unknown	*Bracalba hesperia* Burks ,sp. n.
–	Mesoscutellum without median grooves (Fig. 43). Middle mandibular tooth tiny, much smaller than the other two (Figs 16, 26, 45). Clypeus without median carina. Female: S5 with or without carinae	13
13	Mesoscutellum sparsely foveolate, with broad interspaces (Fig. 60). Female: S5 with longitudinal carinae (Fig. 61)	*Bracalba tricorata* Burks, sp. n.
–	Mesoscutellum densely foveolate (interspaces very narrow) except sometimes a small smooth antero-median area (Figs 14, 24, 43). Female: S5 finely foveolate, without longitudinal carinae (Figs 17, 46)	14
14	Metascutellum trapezoidal in dorsal view, with straight lateral margins (Fig. 43). Female: Antenna with seven apical segments bearing large ventral sensilla, forming a more or less distinct 7-segmented club that includes A6	*Bracalba pinnula* Burks, sp. n.
–	Metascutellum in dorsal view irregularly subrectangular or cushion-like, with irregular margins (Figs 14, 24). Female: Antenna with large ventral sensilla on only the six apical segments, these expanded into a 6-segmented club that is distinct from A6 (Figs 14–15)	15
15	Mesoscutellum with fine wrinkles or carinae in interspaces between foveolae (Fig. 14). Female: T6 broader than long (Fig. 14)	*Bracalba clavata* Burks, sp. n.
–	Mesoscutellum with smooth interspaces between foveolae (Fig. 24); occasionally smaller foveolae may be present in the interspaces, or the interspaces themselves could be raised to form large wrinkles. Female: T6 longer than broad (Fig. 24)	*Bracalba intermedia* Burks, sp. n.

### *Bracalba cuneata* Species Group

#### Bracalba cuneata

Dodd

urn:lsid:zoobank.org:act:10EC007E-A303-4847-BDD9-29AF68935211

urn:lsid:biosci.ohio-state.edu:osuc_concepts:4126

http://species-id.net/wiki/Bracalba_cuneata

**Figures 3–6.**
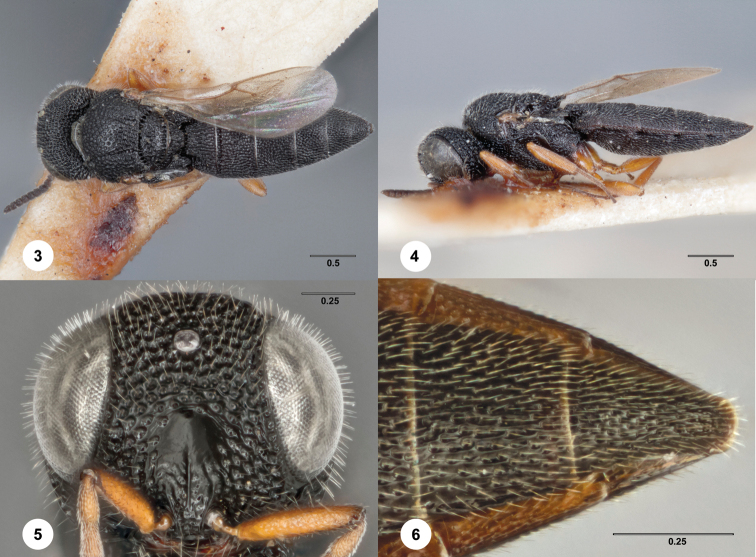
*Bracalba cuneata* Dodd, holotype female (QMBA HY4733). **3** Dorsal habitus **4** Lateral habitus, female (OSUC 238135) **5** Head, anterior view **6** Metasomal sterna 5–6, ventral view. Morphbank^15^

Figures 3–6 Morphbank ^15^

Bracalba cuneata Dodd 1931: 80 (original description); Galloway 1976: 87 (type information); Galloway and Austin 1984: 99 (figure of antenna); Johnson 1992: 354 (catalogued).

##### Description.

*Female*. Body length 3.25–4.00 mm (n=29). Color of antenna beyond radicle: yellowish-brown. Radicle color: same as scape. Number of claval segments with ventral gustatory sensilla: 7. Number of ventral gustatory sensilla on A6: 2.

Ocular setae: short and dense. Frontal depression: smooth dorsally, ventrally with longitudinal carina and with additional carinae arising from medial margins of antennal foramina. Smooth depression extending dorsolaterally from antennal foramen: present. Dorsal clypeal margin: but without other sculpture. Clypeal median carina: absent. Ventral clypeal margin: with a small median point. Mandibular teeth: two, separated by narrow incision. Smooth area obliquely posterior to lateral ocellus: present. Genal sculpture: reticulate-rugose with strong dorsoventral carinae. Mandibular color: dark basally and at teeth, becoming lighter reddish brown between these areas.

Dorsal pronotal area: not set off by carina ventrally. Anterolateral corner of dorsal pronotal area: truncate anteriorly. Sculpture of posteromedian area of mesoscutum: foveolate with broad longitudinal septa. Lateral margin of dorsal axillar area: triangularly expanded or with posterior tooth, broadest posteriorly. Mesoscutellar sculpture: densely foveolate. Metascutellum in dorsal view: trapezoidal with broad apex. Dorsal surface of metascutellum: apex protruding dorsally. Femoral depression: crossed by rounded carinae. Anterior corner of lateral propodeal carina: flat, without tooth. Posteromedial corner of lateral propodeal area: protruding posteriorly. Leg color: yellowish-brown except for dorsal external part of coxae and sometimes tarsomeres 2-5.

Metasoma color: black to dark reddish brown. Median lobe of T1: with 7 or more longitudinal carinae, or with median smooth area interrupting carinae. Metasoma at middle of T4: without bend. T5 median carina: absent. Longitudinal sculptural septa on T5: weak, blunt and hardly raised. Transverse sculptural septa on T5: about as strong as the longitudinal septa. T5 setae: directed posteriorly, arising from anterior edge of sculptural mesh. T6: as broad or broader than long. T6 laterotergite: overlapping S6. S4 median carina: absent. Transverse sculptural septa on S5: weak or absent, much weaker than the longitudinal septa. S5 setae: directed posteriorly, arising from anterior edge of sculptural mesh. Lateral carinae of S6: absent. Apex of S6: without notch.

*Male*. Body length 3.12–3.80 mm (n=15). Flagellomere length: A3 over 1.5× as long as broad, most others as long or longer than broad. T7: flat and posteriorly truncate.

##### Diagnosis.

*Female*. A6 with 2 large ventral sensilla; 2 mandibular teeth; anterolateral corner of dorsal pronotal area truncate anteriorly; metascutellum trapezoidal in dorsal view, with a broad apex, protruding dorsally; metasomal bend absent; T4–T6 without median carina, S4–S6 without median carina; T6 broader than long; S6 without apical notch.

##### Link to distribution map.

http://hol.osu.edu/map-full.html?id=4126

**Associations**. Collected near flowering *Scaevola spinescens* R. Br.: [Asterales: Goodeniaceae]

##### Material examined.

Holotype, female: AUSTRALIA: QLD, Chinchilla, 8.II.1928, A. P. Dodd, QMBA HY4733 (deposited in QMBA). Paratypes: AUSTRALIA: 2 females, OSUC 238448-238449 (ANIC). Other material: AUSTRALIA: 32 females, 20 males, OSUC 238516 (BMNH); OSUC 238120, 238123-238128, 238130-238153, 238159-238166, 238171-238173, 238176, 238180-238181, 238188, 238201-238202 (CNCI); OSUC 376915 (MCZC); OSUC 238451, QM Reg. No. T35161 (QDPC); OSUC 231779 (WINC).

##### Comments.

*Bracalba cuneata* is the most commonly collected species of its genus. Our concept of this speciesincludes some slight variation in metascutellar length and pronotal sculpture. This variation included many intermediates and did not correlate with other characters or with collection locality.

#### Bracalba globosa

Burks sp. n.

urn:lsid:zoobank.org:act:68361C59-4610-4DD5-94CB-0BEB86CA2BB1

urn:lsid:biosci.ohio-state.edu:osuc_concepts:302153

http://species-id.net/wiki/Bracalba_globosa

**Figures 7–11.**
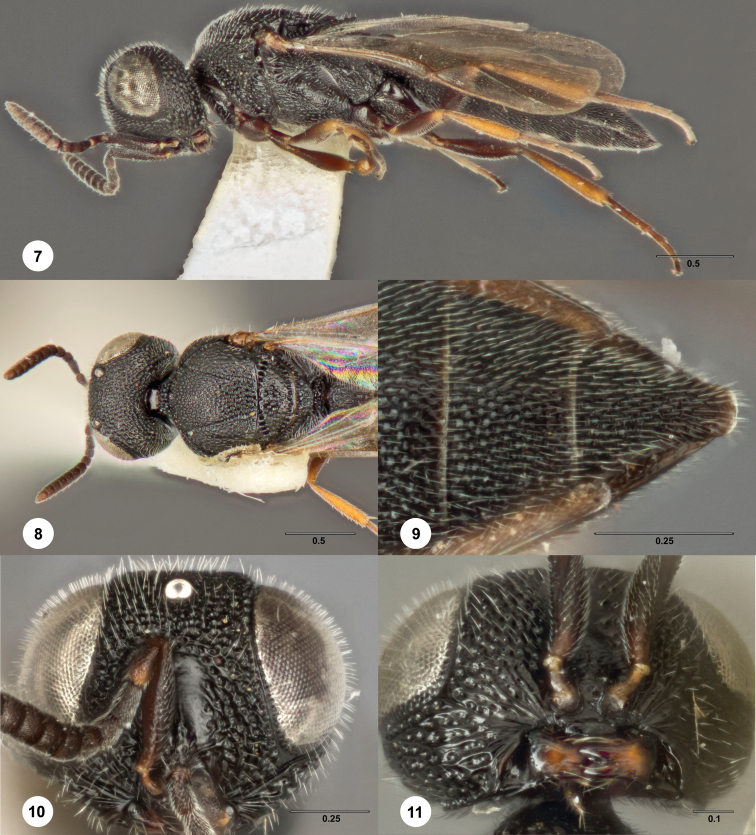
*Bracalba globosa* sp. n.,paratype female (OSUC 148612). **7** Lateral habitus **8** Head and mesosoma, dorsal view **9** Metasomal sterna 5–6, ventral view **11** Head, ventral view, holotype female (OSUC 148701) **10** Head, anterior view. Morphbank^16^

Figures 7–11 Morphbank ^16^

##### Description.

*Female*. Body length 3.12–3.25 mm (n=2). Color of antenna beyond radicle: entirely dark. Radicle color: lighter than scape. Number of claval segments with ventral gustatory sensilla: 7. Number of ventral gustatory sensilla on A6: 2.

Ocular setae: short and dense. Frontal depression: smooth dorsally, torular triangle foveolate with 1 transverse carina extending laterally from inner margin of antennal foramen. Smooth depression extending dorsolaterally from antennal foramen: present. Dorsal clypeal margin: but without other sculpture. Clypeal median carina: absent. Ventral clypeal margin: with a small median point. Mandibular color: dark basally and at teeth, becoming lighter reddish brown between these areas. Mandibular teeth: two, separated by narrow incision. Smooth area obliquely posterior to lateral ocellus: present. Genal sculpture: reticulate-rugose without any strong carinae.

Dorsal pronotal area: not set off by carina ventrally. Anterolateral corner of dorsal pronotal area: weakly rounded anteriorly. Sculpture of posteromedian area of mesoscutum: foveolate with slightly stronger longitudinal septa. Lateral margin of dorsal axillar area: triangularly expanded or with posterior tooth, broadest posteriorly. Mesoscutellar sculpture: densely foveolate with slightly stronger longitudinal septa. Metascutellum in dorsal view: trapezoidal with broad apex. Dorsal surface of metascutellum: apex protruding dorsally. Femoral depression: crossed by rounded carinae. Leg color: dark except for tibiae, 2nd trochanter, and tarsomeres 2-5. Anterior corner of lateral propodeal carina: flat, without tooth. Posteromedial corner of lateral propodeal area: protruding posteriorly.

Metasoma color: black to dark reddish brown. Median lobe of T1: with a set of rugae that merge with one another. Metasoma at middle of T4: without bend. Posterolateral margins of metasomal terga: without protrusions. T5 median carina: absent. Longitudinal sculptural septa on T5: weak, blunt and hardly raised. Transverse sculptural septa on T5: about as strong as the longitudinal septa. T5 setae: directed posteriorly, arising from anterior edge of sculptural mesh. T6: as broad or broader than long. T6 laterotergite: overlapping S6. S4 median carina: absent. Transverse sculptural septa on S5: weak or absent, much weaker than the longitudinal septa. S5 setae: directed posteriorly, arising from anterior edge of sculptural mesh. Lateral carinae of S6: absent. Apex of S6: without notch.

*Male*. Body length 2.88 mm (n=1). Flagellomere length: A3 over 1.5× as long as broad, most others as long or longer than broad. T7: flat and posteriorly truncate.

##### Diagnosis.

*Female*. A6 with 2 large ventral sensilla; 2 mandibular teeth; anterolateral corner of dorsal pronotal area weakly rounded anteriorly; metascutellum trapezoidal with a broad apex, extending dorsally; metasomal bend absent; T4–T6 without median carina, S4–S6 without median carina; T6 broader than long; S6 without apical notch. *Bracalba globosa* is very similar to *Bracalba cuneata*, but differs mainly in mesosomal shape and sculpture. The metascutellum also differs subtly between most specimens of the two species, being smaller in most *Bracalba globosa*. *Bracalba plana* is also very similar to *Bracalba globosa*, but has a very different mesosomal shape.

##### Etymology.

Latin adjective, named after the convex mesoscutum of most specimens in this species.

##### Link to distribution map.

http://hol.osu.edu/map-full.html?id=302153

##### Material examined.

Holotype, female: AUSTRALIA: WA, Kalbarri National Park, 12.XII-19.XII.1986, malaise trap/pan trap, J. S. Noyes, OSUC 148701 (deposited in WAMP). Paratype: AUSTRALIA: 1 female, OSUC 148612 (CNCI).

#### Bracalba plana

Burks sp. n.

urn:lsid:zoobank.org:act:DF77087E-2227-4528-854C-9EE7F7BC7AF1

urn:lsid:biosci.ohio-state.edu:osuc_concepts:302159

http://species-id.net/wiki/Bracalba_plana

**Figures 12–13.**
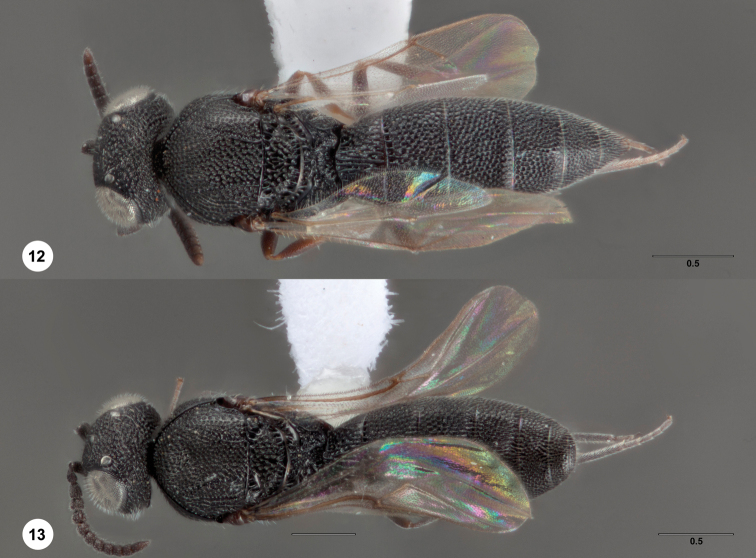
*Bracalba plana* sp. n.,holotype female (OSUC 230804). **12** Dorsal habitus, paratype male (OSUC 148706) **13** Dorsal habitus. Morphbank^17^

Figures 12–13 Morphbank ^17^

##### Description.

*Female*. Body length 2.75–3.13 mm (n=3). Color of antenna beyond radicle: mostly dark, ventral parts of pedicel apex, A4-A12 variably lighter. Radicle color: lighter than scape. Number of claval segments with ventral gustatory sensilla: 7. Number of ventral gustatory sensilla on A6: 2.

Ocular setae: short and dense. Frontal depression: with many irregularly transverse rugae. Smooth depression extending dorsolaterally from antennal foramen: present. Dorsal clypeal margin: absent between antennal foramina. Clypeal median carina: absent. Ventral clypeal margin: with a small median point. Mandibular color: dark basally and at teeth, becoming lighter reddish brown between these areas. Mandibular teeth: two, separated by narrow incision. Smooth area obliquely posterior to lateral ocellus: present. Genal sculpture: deeply reticulate-rugose with some septa much stronger than others, forming distinct rows differing in height.

Dorsal pronotal area: not set off by carina ventrally. Anterolateral corner of dorsal pronotal area: weakly rounded anteriorly. Sculpture of posteromedian area of mesoscutum: densely foveolate. Lateral margin of dorsal axillar area: triangularly expanded or with posterior tooth, broadest posteriorly. Mesoscutellar sculpture: sparsely foveolate, with large smooth interspaces. Metascutellum in dorsal view: semicircular. Dorsal surface of metascutellum: flat. Femoral depression: centrally smooth, peripherally foveolate. Leg color: coxae dark, leg becoming gradually lighter apically. Anterior corner of lateral propodeal carina: flat, without tooth. Posteromedial corner of lateral propodeal area: protruding posteriorly.

Metasoma color: black to dark reddish brown. Median lobe of T1: with 7 or more longitudinal carinae. Metasoma at middle of T4: without bend. Posterolateral margins of metasomal terga: without protrusions. T5 median carina: absent. Longitudinal sculptural septa on T5: weak, blunt and hardly raised. Transverse sculptural septa on T5: about as strong as the longitudinal septa. T5 setae: directed posteriorly, arising from anterior edge of sculptural mesh. T6: as broad or broader than long. T6 laterotergite: overlapped by rim from S6. S4 median carina: absent. Transverse sculptural septa on S5: about as strong as the longitudinal septa. S5 setae: directed posteriorly, arising from anterior edge of sculptural mesh. Lateral carinae of S6: absent. Apex of S6: without notch.

*Males*. Body length 2.75–3.00 mm (n=2). Flagellomere length: A3 1–1.2× as long as broad, most others transverse. T7: flat and posteriorly truncate.

##### Diagnosis.

*Female*. A6 with 2 large ventral sensilla; 2 mandibular teeth; anterolateral corner of dorsal pronotal area weakly rounded anteriorly; metascutellum trapezoidal with a broad apex, flat dorsally; metasomal bend absent; T4–T6 without median carina, S4–S6 without median carina; T6 broader than long; S6 without apical notch. This species is very similar to *Bracalba globosa*, but differs mainly in having a shorter, more flat metascutellum and shorter flagellomeres in males.

##### Etymology.

Latin adjective, referring to the flat mesoscutum, mesoscutellum, and metascutellum of most specimens of this species.

##### Link to distribution map.

http://hol.osu.edu/map-full.html?id=302159

##### Material examined.

Holotype, female: AUSTRALIA: NT, Centre for Zone Research (CSIRO), Alice Springs, XI-1992, malaise trap, Austin & Dangerfield, OSUC 230804 (deposited in SAMA). Paratypes: AUSTRALIA: 5 females, 3 males, OSUC 148706, 238115, 238168, 238170 (CNCI); OSUC 230802-230803, 230805-230806 (WINC).

##### Comments.

Most specimens have a flat mesoscutum and mesoscutellum, but these features were not constant within this species nor in others of the genus.

### *Bracalba laminata* species group

#### Bracalba clavata

Burks sp. n.

urn:lsid:zoobank.org:act:C75BF55D-ADB0-417C-8AF3-EE4E46370C15

urn:lsid:biosci.ohio-state.edu:osuc_concepts:302166

http://species-id.net/wiki/Bracalba_clavata

**Figures 14–18.**
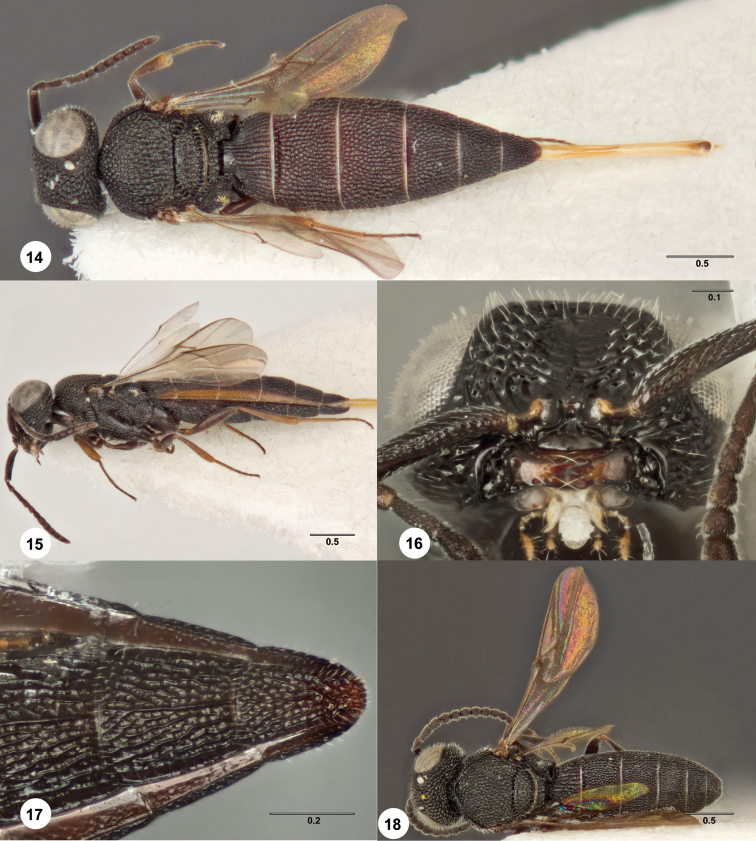
*Bracalba clavata* sp. n.,holotype female (OSUC 384555). **14** Dorsal habitus **15** Lateral habitus, paratype female (OSUC 384558) **16** Head, ventral view, paratype female (OSUC 384559) **17 **Metasomal sterna 5–6, ventral view, paratype male (OSUC 384556) **18** Dorsal habitus. Morphbank^18^

Figures 14–18 Morphbank ^18^

##### Description.

*Female*. Body length 3.25–3.38 mm (n=2). Color of antenna beyond radicle: mostly dark, ventral parts of pedicel apex, A4-A12 variably lighter. Radicle color: base lighter than scape. Number of claval segments with ventral gustatory sensilla: 6. Number of ventral gustatory sensilla on A6: 0.

Ocular setae: short and dense. Frontal depression: with many irregularly transverse rugae, or smooth dorsally, ventrally with oblique carinae converging on a longitudinal ruga. Smooth depression extending dorsolaterally from antennal foramen: present. Dorsal clypeal margin: forming a complete connection between antennal foramina medially. Clypeal median carina: absent. Ventral clypeal margin: with a small median point. Mandibular color: dark basally and at teeth, becoming lighter reddish brown between these areas. Mandibular teeth: three, but middle tooth tiny. Smooth area obliquely posterior to lateral ocellus: present. Genal sculpture: deeply reticulate-rugose with some septa much stronger than others, forming distinct rows differing in height.

Dorsal pronotal area: not set off by carina ventrally. Anterolateral corner of dorsal pronotal area: weakly rounded anteriorly. Sculpture of posteromedian area of mesoscutum: foveolate with broad longitudinal septa. Lateral margin of dorsal axillar area: triangularly expanded or with posterior tooth, broadest posteriorly. Mesoscutellar sculpture: densely foveolate but with smooth central area. Metascutellum in dorsal view: strongly transverse, subrectangular. Dorsal surface of metascutellum: flat, or convex. Femoral depression: centrally smooth, peripherally foveolate. Leg color: coxae, femora (aside from their apices), and at least the last two tarsomeres dark, otherwise yellowish brown. Anterior corner of lateral propodeal carina: flat, without tooth. Posteromedial corner of lateral propodeal area: not protruding posteriorly.

Metasoma color: black to dark reddish brown. Median lobe of T1: with 7 or more longitudinal carinae. Metasoma at middle of T4: with very weak bend. Posterolateral margins of metasomal terga: with tooth-like protrusions. T5 median carina: absent. Longitudinal sculptural septa on T5: strong, sharply raised. Transverse sculptural septa on T5: about as strong as the longitudinal septa. T5 setae: directed posteriorly, arising from anterior edge of sculptural mesh. T6: as broad or broader than long. T6 laterotergite: overlapping S6. S4 median carina: present. Transverse sculptural septa on S5: weak or absent, much weaker than the longitudinal septa. S5 setae: directed posteriorly, arising from anterior edge of sculptural mesh. Lateral carinae of S6: absent. Apex of S6: without notch.

*Male*. Body length 2.75–3.25 mm (n=5). Flagellomere length: A3 over 1.5× as long as broad, most others as long or longer than broad. T7: flat and posteriorly truncate.

##### Diagnosis.

*Female*. A6 without large ventral sensilla, separate and much smaller than A7 (therefore only 6 distinct claval segments present); 3 mandibular teeth with the middle tooth much smaller than the others; metascutellum subrectangular and very short; metasomal bend present but very weak; sculpture posterior to metasomal bend with longitudinal and transverse septa of about equal strength and hardly different from those anterior to the bend; T4–T6 without longitudinal carina; S4–S6 with longitudinal carina; T6 about as long as broad; S6 without apical notch. This species is very similar to *Bracalba pinnula* and to some species without a T4 bend. It differs in the unusual antenna, short metascutellum, and in its shorter wings that reach only to the middle of T4.

##### Etymology.

Latin adjective meaning “clavate,” named for the clavate antenna with its distinct separation between the 6-segmented club and the basal flagellomeres.

##### Link to distribution map.

http://hol.osu.edu/map-full.html?id=302166

##### Material examined.

Holotype, female: AUSTRALIA: SA, Brookfield Conservation Park, 34°21'S, 139°29'E, 24.XI–26.XI.1992, yellow pan trap, I. Naumann & J. C. Cardale, OSUC 384555 (deposited in ANIC). Paratypes: AUSTRALIA: 2 females, 6 males, OSUC 230820, 367520, 384556, 384558-384559, 384564-384566 (ANIC).

#### Bracalba hesperia

Burks sp. n.

urn:lsid:zoobank.org:act:1D575B7A-FD40-4392-B7A5-B0EF403B0168

urn:lsid:biosci.ohio-state.edu:osuc_concepts:302154

http://species-id.net/wiki/Bracalba_hesperia

**Figures 19–23.**
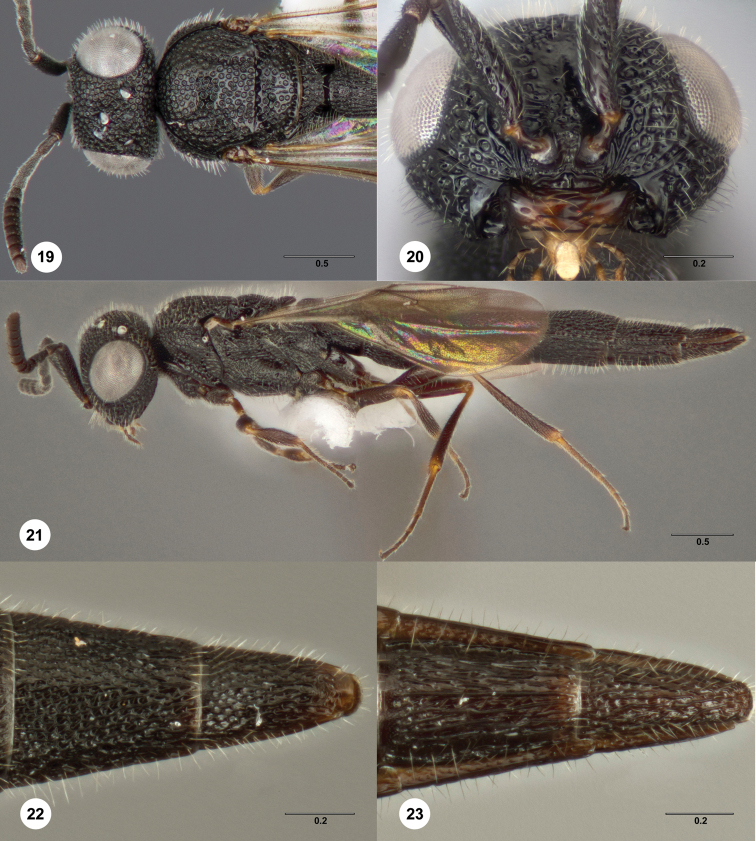
*Bracalba hesperia* sp. n.,holotype female (OSUC 148713). **19** Head and mesosoma, dorsal view **20** Head, ventral view **21** Lateral habitus; **22** Metasomal terga 5–6, dorsal view **23** Metasomal sterna 5–6, ventral view. Morphbank^19^

Figures 19–23 Morphbank ^19^

##### Description.

*Female*. Body length 5.76–6.06 mm (n=2). Color of antenna beyond radicle: entirely dark. Radicle color: same as scape. Number of claval segments with ventral gustatory sensilla: 7. Number of ventral gustatory sensilla on A6: 1.

Ocular setae: short and sparse. Frontal depression: smooth dorsally, torular triangle foveolate with transverse carinae lateral to this area. Smooth depression extending dorsolaterally from antennal foramen: present. Dorsal clypeal margin: wrinkle-like with a median peak. Clypeal median carina: present. Ventral clypeal margin: with a small median point. Mandibular color: dark basally and at teeth, becoming lighter reddish brown between these areas. Mandibular teeth: three of roughly equal size. Smooth area obliquely posterior to lateral ocellus: present. Genal sculpture: reticulate-rugose without any strong carinae.

Dorsal pronotal area: not set off by carina ventrally. Anterolateral corner of dorsal pronotal area: weakly rounded anteriorly. Sculpture of posteromedian area of mesoscutum: densely foveolate. Lateral margin of dorsal axillar area: with a semicircular expansion, broadest near midlength. Mesoscutellar sculpture: densely foveolate with one or two median longitudinal channels. Metascutellum in dorsal view: trapezoidal with broad apex. Dorsal surface of metascutellum: convex. Femoral depression: centrally smooth, peripherally foveolate. Leg color: dark except for trochanters, tips of femora and tibiae, and tarsomeres 1-4. Anterior corner of lateral propodeal carina: flat, without tooth. Posteromedial corner of lateral propodeal area: protruding posteriorly.

Metasoma color: black to dark reddish brown. Median lobe of T1: with 7 or more longitudinal carinae. Metasoma at middle of T4: with metasomal bend and abrupt transition in sculpture. Posterolateral margins of metasomal terga: without protrusions. T5 median carina: absent. or present. Longitudinal sculptural septa on T5: weak, blunt and hardly raised. Transverse sculptural septa on T5: about as strong as the longitudinal septa. T5 setae: directed posteriorly, arising from anterior edge of sculptural mesh. T6: longer than broad. T6 laterotergite: overlapped by rim from S6. S4 median carina: absent. Transverse sculptural septa on S5: weak or absent, much weaker than the longitudinal septa. S5 setae: not directed posteriorly, arising from center of sculptural mesh. Lateral carinae of S6: absent. Apex of S6: without notch.

*Male*. unknown.

##### Diagnosis.

*Female*. A6 with 1 large ventral sensillum; 3 mandibular teeth of approximately equal length; metascutellum broadly trapezoidal with a broad and slightly concave apex; metasomal bend present but weak; sculpture posterior to metasomal bend with transverse septa about as strong as the longitudinal septa; T4–T6 and S4–S6 without median carina; T6 longer than broad; S6 without apical notch. This species is similar to *Bracalba tridentata*, but in that species S6 has a strong apical notch. It is also near *Bracalba nigrescens*, but no complete female specimens of that species are known.

##### Etymology.

Latin adjective, referring to the geographic distribution of this species.

##### Link to distribution map.

http://hol.osu.edu/map-full.html?id=302154

##### Material examined.

Holotype, female: AUSTRALIA: WA, 3km W Walpole, Keystone Road, 34°59.01'S, 116°40.76'E, no date, yellow pan trap, George, Hawks & Munro, OSUC 148713 (deposited in WAMP). Paratypes: AUSTRALIA: 2 females, OSUC 148702, 238154 (CNCI).

##### Comments.

Some singleton specimens similar to *Bracalba hesperia* have been examined but left undescribed, most collected from Western Australia. They all exhibit a stronger metasomal bend and stronger longitudinal sculpture posterior to the bend than in *Bracalba hesperia*, but are variable in these and other characters. Together these specimens and *Bracalba hesperia* may represent members of a species complex. Some specimens within this complex possess longitudinal median grooves on the mesoscutellum, but this character may be variable within species.

#### Bracalba intermedia

Burks sp. n.

urn:lsid:zoobank.org:act:D1FA6338-2827-4D30-AF06-849208F4A6E6

urn:lsid:biosci.ohio-state.edu:osuc_concepts:302155

http://species-id.net/wiki/Bracalba_intermedia

**Figures 24–27.**
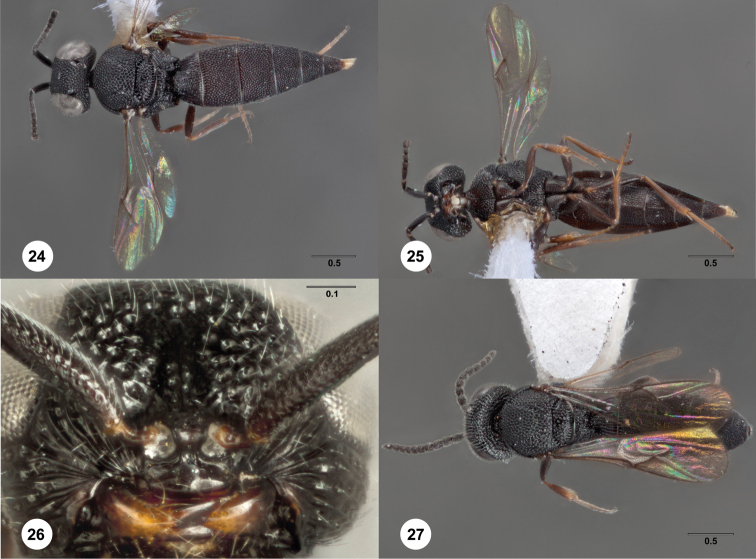
*Bracalba intermedia* sp. n.,paratype female (OSUC 180713). **24** Dorsal habitus **25 **Ventral habitus, paratype female (OSUC 148707) **26** Head, ventral view, paratype male (OSUC 227576) **27** Dorsal habitus. Morphbank^20^

Figures 24–27 Morphbank ^20^

##### Description.

*Female*. Body length 3.25–3.88 mm (n=7). Color of antenna beyond radicle: entirely dark. Radicle color: lighter than scape. Number of claval segments with ventral gustatory sensilla: 6. Number of ventral gustatory sensilla on A6: 0.

Ocular setae: short and dense. Frontal depression: medially smooth, torular triangle sparsely foveolate, or foveolate dorsally, torular triangle foveolate, areas lateral to it with transverse carinae. Smooth depression extending dorsolaterally from antennal foramen: absent. Dorsal clypeal margin: bordering antennal foramina, absent between them. Clypeal median carina: absent. Ventral clypeal margin: convex. Mandibular color: dark basally and at teeth, becoming lighter reddish brown between these areas. Mandibular teeth: three, but middle tooth tiny. Smooth area obliquely posterior to lateral ocellus: present. Genal sculpture: reticulate-rugose without any strong carinae.

Dorsal pronotal area: not set off by carina ventrally. Anterolateral corner of dorsal pronotal area: weakly rounded anteriorly. Sculpture of posteromedian area of mesoscutum: foveolate with slightly stronger longitudinal septa. Lateral margin of dorsal axillar area: triangularly expanded or with posterior tooth, broadest posteriorly. Mesoscutellar sculpture: densely foveolate with slightly stronger longitudinal septa. Metascutellum in dorsal view: strongly transverse, subrectangular. Dorsal surface of metascutellum: flat. Femoral depression: centrally smooth, peripherally foveolate. Leg color: coxae, femora (aside from their apices), and at least the last two tarsomeres dark, otherwise yellowish brown. Anterior corner of lateral propodeal carina: flat, without tooth. Posteromedial corner of lateral propodeal area: protruding posteriorly.

Metasoma color: black to dark reddish brown. Median lobe of T1: with a set of rugae that merge with one another. Metasoma at middle of T4: without bend. Posterolateral margins of metasomal terga: without protrusions. T5 median carina: absent. Longitudinal sculptural septa on T5: weak, blunt and hardly raised. Transverse sculptural septa on T5: about as strong as the longitudinal septa. T5 setae: directed posteriorly, arising from anterior edge of sculptural mesh. T6: longer than broad. T6 laterotergite: overlapping S6. S4 median carina: present. Transverse sculptural septa on S5: weak or absent, much weaker than the longitudinal septa. S5 setae: directed posteriorly, arising from anterior edge of sculptural mesh. Lateral carinae of S6: absent. Apex of S6: without notch.

*Male*. Body length 2.62–3.25 mm (n=18). Flagellomere length: A3 over 1.5× as long as broad, most others as long or longer than broad. T7: flat and posteriorly truncate.

##### Diagnosis.

*Female*. A6 without large ventral sensilla; 3 mandibular teeth with middle tooth much smaller than the others; metascutellum broad and subrectangular, very short; metasomal bend absent; T4–T6 without median carina, S4–S6 with at least a vague median carina; T6 longer than broad; S6 without apical notch. The antenna and short metascutellum help distinguish it from similar species. It is very similar to *Bracalba pinnula*, but differs in having a much shorter metascutellum. It is also very similar to *Bracalba clavata*, but has no distinct metasomal bend and has a longer T6 in females.

##### Etymology.

Latin adjective, referring to its unusual mixture of characters.

##### Link to distribution map.

http://hol.osu.edu/map-full.html?id=302155

##### Material examined. 

Holotype, female: AUSTRALIA: WA, via Dwellingup, Yarragil 4P Catchment, 18.II-25.II.1981, malaise trap, A. Postle, OSUC 230811 (deposited in QMBA). Paratypes: AUSTRALIA: 8 females, 23 males, OSUC 148705, 148707-148708, 180713, 238105-238109, 238111, 238179, 238182, 238184-238186 (CNCI); OSUC 230809-230810, 230812, 230814, 238450 (QDPC); OSUC 227572-227578, 230801, 238454, 238457, 384567 (WINC).

##### Comments.

*Bracalba intermedia* lacks a metasomal bend, but the three mandibular teeth, elongate metasoma, and sculptural features suggest that it belongs in the *laminata* group. Specimens from the eastern localities tend to be slightly smaller and have some reduction in sculpture, but are retained in our concept of this species.

#### Bracalba laminata

Dodd

urn:lsid:zoobank.org:act:4C6F2E7E-3E8C-4973-AC72-D02632852D69

urn:lsid:biosci.ohio-state.edu:osuc_concepts:4127

http://species-id.net/wiki/Bracalba_laminata

**Figures 28–31.**
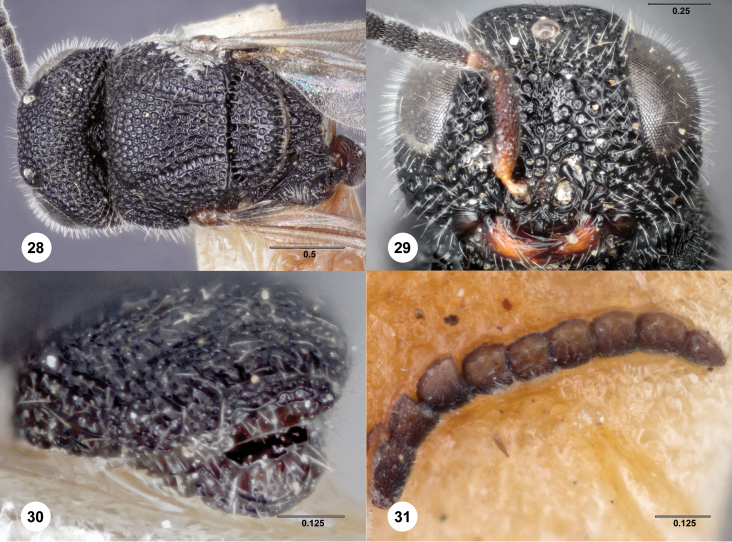
*Bracalba laminata* Dodd, allotype male (QMBA HY4732A). **28** Head and mesosoma, dorsal view **29** Head, anterior view **30** Metasomal terga 6–8, posterior view, holotype female (QMBA HY4732H) **31** Antenna, ventral view. Morphbank^21^

Figures 28–31 Morphbank ^21^

Bracalba laminata Dodd 1931: 78 (original description); Galloway 1976: 88 (type information); Johnson 1992: 354 (catalogued).

##### Description.

*Female*. Number of claval segments with ventral gustatory sensilla: 7. Number of ventral gustatory sensilla on A6: 2.

*Male*. Body length = 3.38 mm (n=1). Flagellomere length: A3 over 1.5× as long as broad, most others as long or longer than broad. Ocular setae: long and dense. Frontal depression: densely foveolate. Smooth depression extending dorsolaterally from antennal foramen: present. Dorsal clypeal margin: absent between antennal foramina. Clypeal median carina: absent. Ventral clypeal margin: with a small median point. Mandibular teeth: three, but middle tooth tiny. Smooth area obliquely posterior to lateral ocellus: present. Genal sculpture: deeply reticulate-rugose with some septa much stronger than others, forming distinct rows differing in height. Mandibular color: dark basally and at teeth, becoming lighter reddish brown between these areas.

Dorsal pronotal area: not set off by carina ventrally. Anterolateral corner of dorsal pronotal area: truncate anteriorly. Sculpture of posteromedian area of mesoscutum: foveolate with slightly stronger longitudinal septa. Lateral margin of dorsal axillar area: with a semicircular expansion, broadest near midlength. Mesoscutellar sculpture: densely foveolate with a longitudinal carina. Metascutellum in dorsal view: elongate-trapezoidal but with incised apex. Dorsal surface of metascutellum: apex protruding dorsally. Femoral depression: crossed by rounded carinae. Anterior corner of lateral propodeal carina: flat, without tooth. Posteromedial corner of lateral propodeal area: protruding posteriorly. T7: arched and posteriorly concave.

##### Diagnosis.

Males: Lateral margin of dorsal axillar area semicircularly expanded; mesosoma broadly truncate anteriorly; mesoscutellum with median carina; metascutellum elongate-trapezoidal with incised apex.

##### Link to distribution map.

http://hol.osu.edu/map-full.html?id=4127

##### Material Examined. 

Holotype, female: AUSTRALIA: QLD, Gogango, 40mi W Rockhampton, III-1928, A. P. Dodd, QMBA HY4732H (deposited in QMBA). Allotype: AUSTRALIA: 1 male, QMBA HY4732A (QMBA). Other material: AUSTRALIA: 3 males, OSUC 365210-365212 (UQIC).

##### Comments.

Only the antenna and a few legs of the female holotype of *Bracalba laminata* remain. The syntopic allotype male strongly resembles the holotype of *Bracalba nigrescens*, but differs in the presence of a partial median carina on the mesoscutellum, the anteriorly truncate mesosoma, and denser sculpturing on the thoracic dorsum. No other female specimens are known aside from the holotype. The allotype shares characteristics with *Bracalba hesperia*, *Bracalba magnirubra*, and *Bracalba tridentata*, but differs enough that it cannot definitively be associated with any of these species.

Because the antenna and some legs of the holotype remains, they now represent the holotype. A neotype cannot be properly designated without first requesting that the existing holotype be set aside. This was considered unnecessary, as the antenna clearly belongs to *Bracalba*, based on the number and arrangement of ventral sensilla. Therefore, there is no doubt that the holotype agrees with our concept of *Bracalba*. There is also no reason to conclude that the allotype male would be from a different species than the female, based on its morphology compared with Dodd’s (1931) description of the now lost parts of the female’s body.

#### Bracalba magnirubra

Burks sp. n.

urn:lsid:zoobank.org:act:48277EF5-2342-478C-8BDE-3B3F2AF0790E

urn:lsid:biosci.ohio-state.edu:osuc_concepts:302156

http://species-id.net/wiki/Bracalba_magnirubra

**Figures 32–35.**
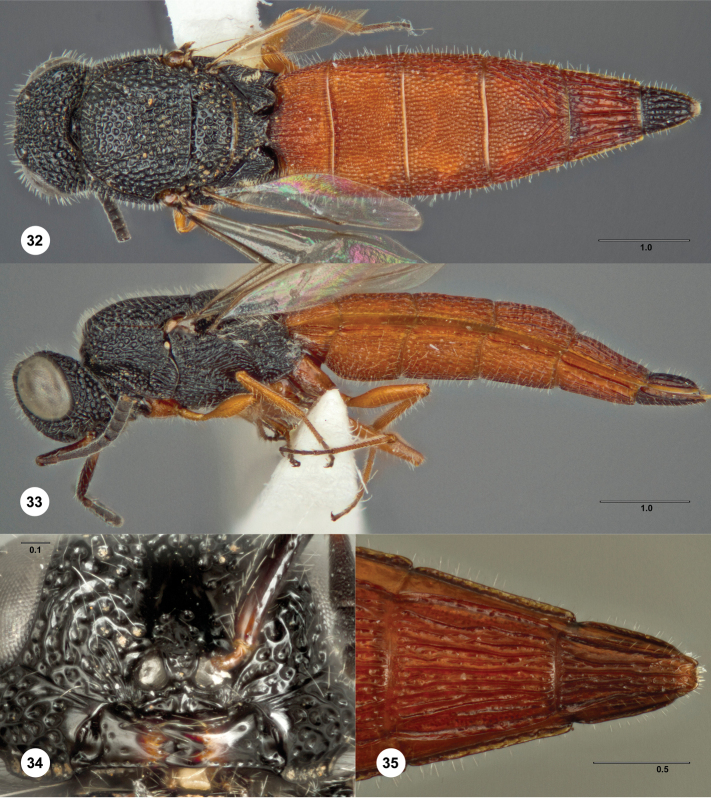
*Bracalba magnirubra* sp. n.,paratype female (OSUC 238178). **32** Dorsal habitus **34** Head, ventral view, holotype female (OSUC 148715) **33** Lateral habitus, paratype female (OSUC 238112) **35** Metasomal sterna 5–6, ventral view. Morphbank^22^

Figures 32–35 Morphbank ^22^

##### Description.

*Female*. Body length 6.50–6.88 mm (n=3). Color of antenna beyond radicle: mostly dark, extreme base of scape becoming reddish. Radicle color: lighter than scape. Number of claval segments with ventral gustatory sensilla: 7. Number of ventral gustatory sensilla on A6: 1.

Ocular setae: long and sparse. Frontal depression: medially smooth, torular triangle sparsely foveolate. Smooth depression extending dorsolaterally from antennal foramen: present. Dorsal clypeal margin: interrupted by a median areole below interantennal process. Clypeal median carina: present. Ventral clypeal margin: with a small median point. Mandibular color: dark basally and at teeth, becoming lighter reddish brown between these areas. Mandibular teeth: three, but middle tooth tiny. Smooth area obliquely posterior to lateral ocellus: present. Genal sculpture: reticulate-rugose without any strong carinae.

Dorsal pronotal area: not set off by carina ventrally. Anterolateral corner of dorsal pronotal area: protruding anteriorly. Sculpture of posteromedian area of mesoscutum: densely foveolate. Lateral margin of dorsal axillar area: with a semicircular expansion, broadest near midlength. Mesoscutellar sculpture: densely foveolate. Metascutellum in dorsal view: elongate-trapezoidal but with incised apex. Dorsal surface of metascutellum: apex protruding dorsally, or convex. Femoral depression: crossed by rounded carinae. Leg color: reddish but with at least last two tarsomeres dark. Anterior corner of lateral propodeal carina: flat, without tooth. Posteromedial corner of lateral propodeal area: protruding posteriorly.

Metasoma color: reddish, with last segment variably dark. Median lobe of T1: with 7 or more longitudinal carinae. Metasoma at middle of T4: with metasomal bend and abrupt transition in sculpture. Posterolateral margins of metasomal terga: with tooth-like protrusions. T5 median carina: present. Longitudinal sculptural septa on T5: strong, sharply raised. Transverse sculptural septa on T5: weak or absent, much weaker than the longitudinal septa. T5 setae: not directed posteriorly, arising from center of sculptural mesh. T6: longer than broad. T6 laterotergite: overlapping S6. S4 median carina: present. Transverse sculptural septa on S5: weak or absent, much weaker than the longitudinal septa. S5 setae: not directed posteriorly, arising from center of sculptural mesh. Lateral carinae of S6: not meeting apically. Apex of S6: with notch.

*Male*. Body length 4.75–5.00 mm (n=5). Flagellomere length: A3 over 1.5× as long as broad, most others as long or longer than broad. T7: arched and posteriorly concave.

##### Diagnosis.

*Female*. A6 with 1 large ventral sensillum; 3 mandibular teeth with middle tooth smaller than the others; metascutellum trapezoidal and relatively long, with a truncate or slightly incised apex; metasomal bend present; sculpture posterior to metasomal bend with strong longitudinal septa but without transverse septa; T4–T6 and S4–S6 with median carina; T6 longer than broad; S6 with apical notch.

##### Etymology.

Compound adjective using the Latin adjectives magnus and ruber.

##### Link to distribution map.

http://hol.osu.edu/map-full.html?id=302156

##### Material examined.

Holotype, female: AUSTRALIA: SA, 32km N Renmark, Amalia Dam, xeric mallee scrub, MT 4, ROM 2000040, Bookmark Biosphere Reserve, 33°53'S, 140°43'E, 263m, 15.II–15.IV.2000, malaise trap, D. C. Darling, OSUC 148715 (deposited in SAMA). Paratypes: AUSTRALIA: 4 females, 6 males, OSUC 365208, 367506-367507 (ANIC); OSUC 238112, 238117-238119, 238121-238122, 238178 (CNCI).

##### Comments. 

This is the largest-bodied species of *Bracalba* known. It resembles *Bracalba sculptifrons* and *Bracalba sparsa*, but differs from both in a large number of characters.

#### Bracalba nigrescens

(Dodd)

urn:lsid:zoobank.org:act:3692A700-53E1-4B03-8247-BEE9AC332619

urn:lsid:biosci.ohio-state.edu:osuc_concepts:4128

http://species-id.net/wiki/Bracalba_nigrescens

**Figures 36–39.**
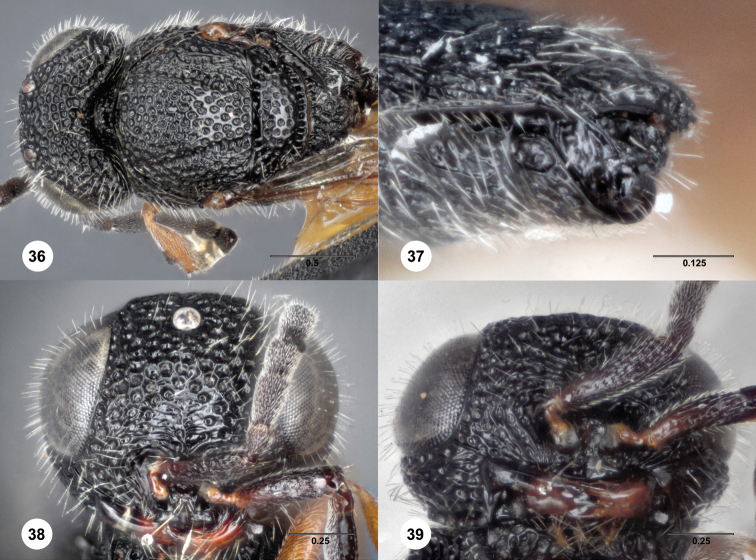
*Bracalba nigrescens* (Dodd) holotype male (B.M. TYPE HYM. 9.499). **36** Head and mesosoma, dorsal view **37** Metasomal terga 6–8, postero-lateral view **38** Head, anterior view **39** Head, ventral view. Morphbank^23^

Figures 36–39 Morphbank ^23^

Chromoteleia nigrescens Dodd 1920: 329 (original description); Masner 1965: 71 (type information).
Bracalba nigrescens (Dodd): Dodd 1931: 80 (generic transfer); Galloway 1976: 88 (type information); Johnson 1992: 354 (catalogued).

##### Description.

*Male*. Body length 3.38 mm (n=1). Flagellomere length: A3 over 1.5× as long as broad, most others as long or longer than broad. Ocular setae: long and dense. Frontal depression: with many irregularly transverse rugae. Smooth depression extending dorsolaterally from antennal foramen: present. Dorsal clypeal margin: absent between antennal foramina. Clypeal median carina: absent. Ventral clypeal margin: with a small median point. Mandibular teeth: three, but middle tooth tiny. Smooth area obliquely posterior to lateral ocellus: present. Genal sculpture: deeply reticulate-rugose with some septa much stronger than others, forming distinct rows differing in height. Mandibular color: mostly reddish brown, dark at teeth.

Dorsal pronotal area: not set off by carina ventrally. Anterolateral corner of dorsal pronotal area: weakly rounded anteriorly. Sculpture of posteromedian area of mesoscutum: sparsely foveolate, with large smooth interspaces. Lateral margin of dorsal axillar area: with a semicircular expansion, broadest near midlength. Mesoscutellar sculpture: sparsely foveolate, with large smooth interspaces. Metascutellum in dorsal view: elongate-trapezoidal but with incised apex. Dorsal surface of metascutellum: apex protruding dorsally. Femoral depression: crossed by rounded carinae. Anterior corner of lateral propodeal carina: flat, without tooth. Posteromedial corner of lateral propodeal area: protruding posteriorly. Leg color: coxae and at least the last three tarsomeres dark, otherwise yellowish. T7: arched and posteriorly concave.

##### Diagnosis.

Males: Lateral margin of dorsal axillar area semicircularly expanded; mesosoma weakly rounded anteriorly; mesoscutellum without median carina; metascutellum elongate-trapezoidal with incised apex.

##### Link to distribution map.

http://hol.osu.edu/map-full.html?id=4128

##### Material examined.

Holotype, male, *Chromoteleia nigrescens*: **AUSTRALIA:** WA, Yallingup, 1.XII-12.XII.1913, R. E. Turner, B.M. TYPE HYM. 9.499 (deposited in BMNH).

##### Comments.

The male holotype of *Bracalba nigrescens* cannot be associated with any known female *Bracalba*. It is very similar to *Bracalba laminata* even though the two species occur on opposite sides of the Australian continent. These species may be closely related to *Bracalba hesperia* and *Bracalba tridentata*.

#### Bracalba parvirubra

Burks sp. n.

urn:lsid:zoobank.org:act:9B615296-D426-4E5E-937A-9AC299199003

urn:lsid:biosci.ohio-state.edu:osuc_concepts:302157

http://species-id.net/wiki/Bracalba_parvirubra

**Figures 40–42.**
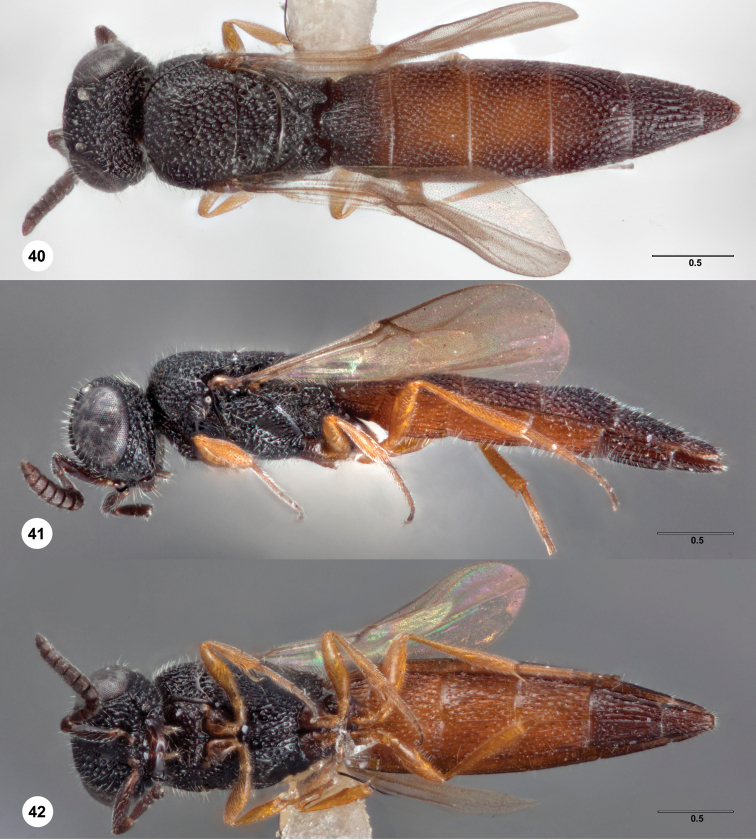
*Bracalba parvirubra* sp. n.,holotype female (OSUC 238194). **40** Dorsal habitus **41 **Lateral habitus **42** Ventral habitus. Morphbank^24^

Figures 40–42 Morphbank ^24^

##### Description.

*Female*. Body length 3.38–3.63 mm (n=3). Color of antenna beyond radicle: mostly dark, extreme base of scape becoming reddish. Radicle color: same as scape. Number of claval segments with ventral gustatory sensilla: 7. Number of ventral gustatory sensilla on A6: 1.

Ocular setae: long and sparse. Frontal depression: smooth dorsally, torular triangle foveolate, areas lateral to it smooth. Smooth depression extending dorsolaterally from antennal foramen: present. Dorsal clypeal margin: arched, interrupted by broad median carina. Clypeal median carina: present. Ventral clypeal margin: with a small median point. Mandibular color: dark basally and at teeth, becoming lighter reddish brown between these areas. Mandibular teeth: three, but middle tooth tiny. Smooth area obliquely posterior to lateral ocellus: present. Genal sculpture: deeply reticulate-rugose with some septa much stronger than others, forming distinct rows differing in height.

Dorsal pronotal area: not set off by carina ventrally. Anterolateral corner of dorsal pronotal area: protruding anteriorly. Sculpture of posteromedian area of mesoscutum: foveolate with slightly stronger longitudinal septa. Lateral margin of dorsal axillar area: with a semicircular expansion, broadest near midlength. Mesoscutellar sculpture: densely foveolate with one or two median longitudinal channels. Metascutellum in dorsal view: elongate-trapezoidal but with incised apex. Dorsal surface of metascutellum: flat, or convex. Femoral depression: crossed by many sharply defined carinae. Leg color: yellowish-brown except for dorsal external part of coxae and sometimes tarsomeres 2-5. Anterior corner of lateral propodeal carina: flat, without tooth. Posteromedial corner of lateral propodeal area: protruding posteriorly.

Metasoma color: centrally reddish with some vague dark intrusions, peripherally becoming black. Median lobe of T1: with 7 or more longitudinal carinae. Metasoma at middle of T4: with metasomal bend and abrupt transition in sculpture. Posterolateral margins of metasomal terga: with tooth-like protrusions. T5 median carina: absent. Longitudinal sculptural septa on T5: strong, sharply raised. Transverse sculptural septa on T5: about as strong as the longitudinal septa. T5 setae: directed posteriorly, arising from anterior edge of sculptural mesh. T6: as broad or broader than long. T6 laterotergite: overlapped by rim from S6. S4 median carina: present. Transverse sculptural septa on S5: weak or absent, much weaker than the longitudinal septa. S5 setae: directed posteriorly, arising from anterior edge of sculptural mesh. Lateral carinae of S6: forming complete peripheral carina. Apex of S6: without notch.

*Male*. unknown.

##### Diagnosis.

*Female*. A6 with 1 large ventral sensillum; 3 mandibular teeth with middle tooth much smaller than the others; metascutellum trapezoidal and strongly narrowing to an incised apex; metasomal bend present; sculpture posterior to metasomal bend with strong longitudinal and transverse septa; T4–T6 without distinct median carina; S4–S6 with median carina; T6 about as broad as long; S6 without apical notch. This species is similar to *Bracalba sculptifrons* and *Bracalba sparsa*, but lacks the S6 notch and frontal carinae.

##### Etymology.

Compound adjective using the Latin adjectives parvus and ruber.

##### Link to distribution map.

http://hol.osu.edu/map-full.html?id=302157

##### Material examined.

Holotype, female: AUSTRALIA: WA, 20km N Denmark, 16.I.1987, J. S. Noyes, OSUC 238194 (deposited in WAMP). Paratypes: AUSTRALIA: 3 females, OSUC 238191, 238193, 238196 (CNCI).

#### Bracalba pinnula

Burks sp. n.

urn:lsid:zoobank.org:act:29EE41F2-7AE8-46F5-AE0C-C9250E6A5771

urn:lsid:biosci.ohio-state.edu:osuc_concepts:302158

http://species-id.net/wiki/Bracalba_pinnula

**Figures 43–46.**
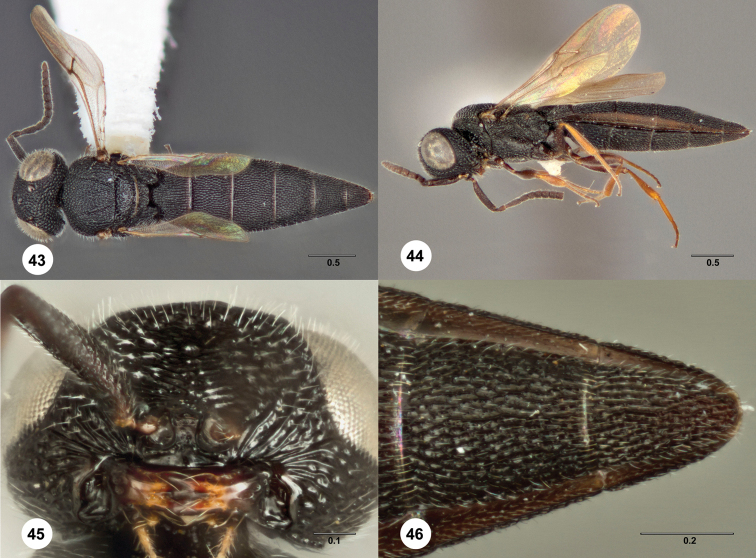
*Bracalba pinnula* sp. n.,paratype female (OSUC 231794). **43** Dorsal habitus **45** Head, ventral view, holotype female (OSUC 231797) **44** Lateral habitus **46** Metasomal sterna 5–6, ventral view. Morphbank^25^

Figures 43–46 Morphbank ^25^

##### Description.

*Female*. Body length 3.37–3.75 mm (n=7). Color of antenna beyond radicle: entirely dark. Radicle color: lighter than scape. Number of claval segments with ventral gustatory sensilla: 7. Number of ventral gustatory sensilla on A6: 1, or 2.

Ocular setae: short and dense. Frontal depression: with many strong transverse carinae, sparsely foveolate at torular triangle. Smooth depression extending dorsolaterally from antennal foramen: present. Dorsal clypeal margin: absent between antennal foramina. Clypeal median carina: absent. Ventral clypeal margin: with a small median point. Mandibular color: dark basally and at teeth, becoming lighter reddish brown between these areas. Mandibular teeth: three of roughly equal size. Smooth area obliquely posterior to lateral ocellus: present. Genal sculpture: deeply reticulate-rugose with some septa much stronger than others, forming distinct rows differing in height.

Dorsal pronotal area: not set off by carina ventrally. Anterolateral corner of dorsal pronotal area: weakly rounded anteriorly. Sculpture of posteromedian area of mesoscutum: densely foveolate. Lateral margin of dorsal axillar area: with a semicircular expansion, broadest near midlength. Mesoscutellar sculpture: densely foveolate. Metascutellum in dorsal view: trapezoidal with broad apex. Dorsal surface of metascutellum: flat, or convex. Femoral depression: centrally smooth, peripherally foveolate. Leg color: coxae and femora dark, but tibiae, tarsi, and sometimes 2nd trochanters yellowish brown. Anterior corner of lateral propodeal carina: flat, without tooth. Posteromedial corner of lateral propodeal area: protruding posteriorly.

Metasoma color: black to dark reddish brown. Median lobe of T1: with a set of rugae that merge with one another. Metasoma at middle of T4: with metasomal bend and abrupt transition in sculpture. Posterolateral margins of metasomal terga: without protrusions. T5 median carina: absent. Longitudinal sculptural septa on T5: weak, blunt and hardly raised. Transverse sculptural septa on T5: about as strong as the longitudinal septa. T5 setae: directed posteriorly, arising from anterior edge of sculptural mesh. T6: as broad or broader than long. T6 laterotergite: overlapping S6. S4 median carina: present. Transverse sculptural septa on S5: about as strong as the longitudinal septa. S5 setae: directed posteriorly, arising from anterior edge of sculptural mesh. Lateral carinae of S6: absent. Apex of S6: without notch.

*Male*. Body length 2.75–3.13 mm (n=4). Flagellomere length: A3 over 1.5× as long as broad, most others as long or longer than broad. T7: flat and posteriorly truncate.

##### Diagnosis.

*Female*. A6 with 1 or 2 large sensilla; 3 mandibular teeth of approximately equal length; metascutellum trapezoidal with a broad and truncate or slightly concave apex; metasomal bend present but very weak; sculpture posterior to metasomal bend with longitudinal and transverse septa of about equal height and hardly differing from those anterior to the bend; T4–T6 and S4-S5 without median carina; S4 sometimes with a slight median carina; T6 longer than broad; S5 with longitudinal septa not stronger than transverse septa; S6 without apical notch. *Bracalba pinnula* has only a very slight metasomal bend, and therefore it can easily be confused with those species that lack the bend. It differs from most of these species in that the fore wings reach to the middle of T4, and from *Bracalba intermedia* in having a long and trapezoidal metascutellum.

##### Etymology.

Latin noun, meaning “a small fin.” This is considered to be a noun in apposition to the generic name.

##### Link to distribution map.

http://hol.osu.edu/map-full.html?id=302158

##### Material examined.

Holotype, female: AUSTRALIA: WA, WA122401, 7km N Stirling Range National Park, 34°19'S, 118°11'E, 900ft, 24.XII.1994, malaise trap, L. S. Kimsey & R. B. Kimsey, OSUC 231797 (deposited in WAMP). Paratypes: AUSTRALIA: 12 females, 11 males, OSUC 149756 (AEIC); OSUC 230822-230824, 367508, 367512-367519 (ANIC); OSUC 148700, 231780-231781 (CNCI); OSUC 238452 (QDPC); OSUC 179086, 179088-179089, 231794-231796 (UCDC).

#### Bracalba propodealis

Burks sp. n.

urn:lsid:zoobank.org:act:F867EC04-0D6C-43C9-8BF8-E134A5C8D488

urn:lsid:biosci.ohio-state.edu:osuc_concepts:302160

http://species-id.net/wiki/Bracalba_propodealis

**Figures 47–50.**
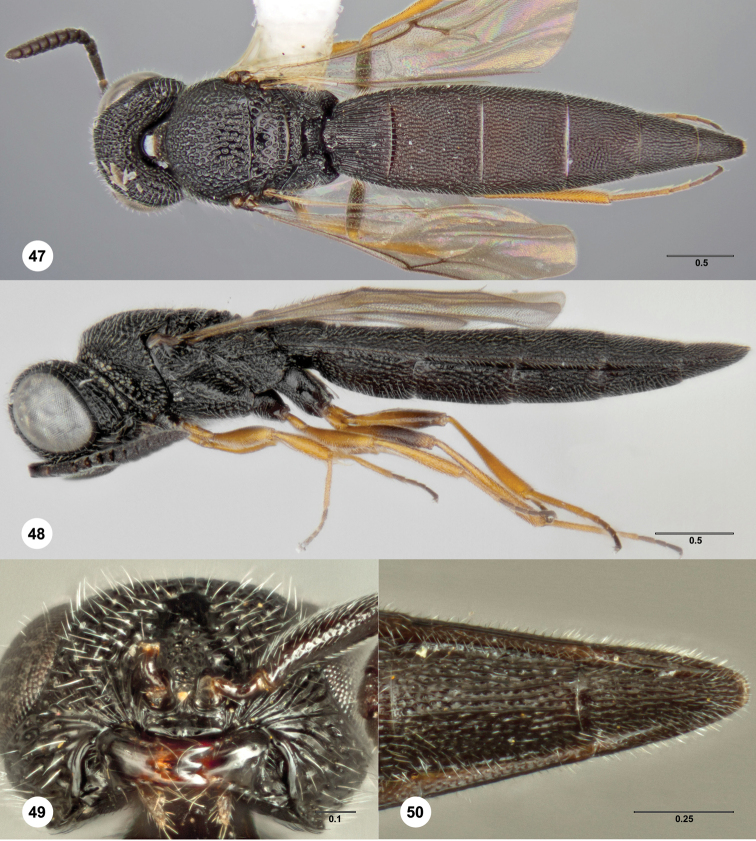
*Bracalba propodealis* sp. n.,holotype female (OSUC 227590). **47** Dorsal habitus, paratype female (OSUC 227591) **48** Lateral habitus, paratype female (OSUC 238190) **49** Head, ventral view **50** Metasomal sterna 5–6, ventral view. Morphbank^26^

Figures 47–50 Morphbank ^26^

##### Description.

*Female*. Body length 3.62–3.88 mm (n=3). Color of antenna beyond radicle: entirely dark. Radicle color: base lighter than scape. Number of claval segments with ventral gustatory sensilla: 7. Number of ventral gustatory sensilla on A6: 1.

Ocular setae: short and dense. Frontal depression: present as a vague smooth triangle. Smooth depression extending dorsolaterally from antennal foramen: present. Dorsal clypeal margin: absent between antennal foramina. Clypeal median carina: absent. Ventral clypeal margin: with a small median point. Mandibular color: dark basally and at teeth, becoming lighter reddish brown between these areas. Mandibular teeth: three, but middle tooth tiny. Smooth area obliquely posterior to lateral ocellus: present. Genal sculpture: deeply reticulate-rugose with some septa much stronger than others, forming distinct rows differing in height.

Dorsal pronotal area: not set off by carina ventrally. Anterolateral corner of dorsal pronotal area: weakly rounded anteriorly. Sculpture of posteromedian area of mesoscutum: foveolate with broad longitudinal septa. Lateral margin of dorsal axillar area: with a semicircular expansion, broadest near midlength. Mesoscutellar sculpture: sparsely foveolate, with large smooth interspaces. Metascutellum in dorsal view: very short, subrectangular. Dorsal surface of metascutellum: convex. Femoral depression: irregularly foveolate but not crossed by carinae. Leg color: coxae, femora (aside from their apices), and at least the last two tarsomeres dark, otherwise yellowish brown. Anterior corner of lateral propodeal carina: with longitudinal tooth-like crest extending dorsally above level of metascutellum. Posteromedial corner of lateral propodeal area: protruding posteriorly.

Metasoma color: black to dark reddish brown. Median lobe of T1: with 7 or more longitudinal carinae. Metasoma at middle of T4: with metasomal bend and abrupt transition in sculpture. Posterolateral margins of metasomal terga: without protrusions. T5 median carina: absent. Longitudinal sculptural septa on T5: weak, blunt and hardly raised. Transverse sculptural septa on T5: about as strong as the longitudinal septa. T5 setae: directed posteriorly, arising from anterior edge of sculptural mesh. T6: longer than broad. T6 laterotergite: overlapping S6. S4 median carina: present. Transverse sculptural septa on S5: about as strong as the longitudinal septa. S5 setae: not directed posteriorly, arising from center of sculptural mesh. Lateral carinae of S6: absent. Apex of S6: without notch.

*Male*. unknown.

##### Diagnosis.

*Female*. A6 with 1 large ventral sensillum; 3 mandibular teeth with the middle tooth much smaller than the others; metascutellum subrectangular, very short and broad; anteromedial margins of lateral propodeal area extending dorsally above the metascutellar surface as distinct protrusions; metasomal bend extremely weak; sculpture posterior to metasomal bend with longitudinal septa stronger than those anterior to the bend, transverse septa as strong or slightly weaker than longitudinal septa; T4–T6 and S6 without median carina, S4-S5 with median carina; S6 without apical notch.

##### Etymology.

An adjective, combining the anatomical term propodeum with the Latin adjectival suffix -alis, referring to its unique propodeum.

##### Link to distribution map.

http://hol.osu.edu/map-full.html?id=302160

##### Material examined.

Holotype, female: AUSTRALIA: WA, Mount Cooke, 28.I-17.II.1991, malaise trap, M. S. Harvey & J. M. Waldock, OSUC 227590 (deposited in WAMP). Paratypes: AUSTRALIA: 2 females, OSUC 238190 (CNCI); OSUC 227591 (WINC).

##### Comments.

The propodeum in this species is unique among the specimens studied. It is very difficult to determine in all examined specimens if the metasomal bend is present or not.

#### Bracalba sculptifrons

Burks sp. n.

urn:lsid:zoobank.org:act:F99233BD-F270-46FF-8E14-9507C07E6D9C

urn:lsid:biosci.ohio-state.edu:osuc_concepts:302161

http://species-id.net/wiki/Bracalba_sculptifrons

**Figures 51–54.**
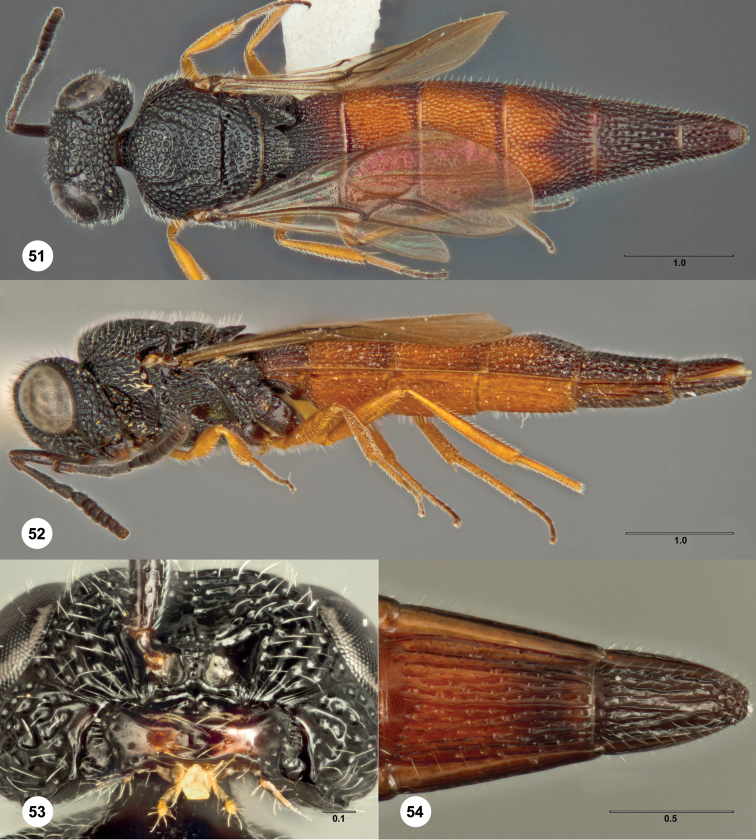
*Bracalba sculptifrons* sp. n.,paratype female (OSUC 148704). **51** Dorsal habitus **53 **Head, ventral view **54** Metasomal sterna 5–6, ventral view, holotype female (OSUC 231799) **52 **Lateral habitus. Morphbank^27^

Figures 51–54 Morphbank ^27^

##### Description.

*Female*. Body length 5.75–6.13 mm (n=2). Color of antenna beyond radicle: entirely dark. Radicle color: same as scape. Number of claval segments with ventral gustatory sensilla: 7. Number of ventral gustatory sensilla on A6: 2.

Ocular setae: short and dense. Frontal depression: with irregular rugae indicating large foveae. Smooth depression extending dorsolaterally from antennal foramen: present. Dorsal clypeal margin: angular, emarginate medially. Clypeal median carina: present. Ventral clypeal margin: with a small median point. Mandibular color: dark basally and at teeth, becoming lighter reddish brown between these areas. Mandibular teeth: three, but middle tooth tiny. Smooth area obliquely posterior to lateral ocellus: present. Genal sculpture: reticulate-rugose with strong dorsoventral carinae.

Dorsal pronotal area: not set off by carina ventrally. Anterolateral corner of dorsal pronotal area: truncate anteriorly. Sculpture of posteromedian area of mesoscutum: densely foveolate. Lateral margin of dorsal axillar area: with a semicircular expansion, broadest near midlength. Mesoscutellar sculpture: densely foveolate. Metascutellum in dorsal view: trapezoidal with broad apex. Dorsal surface of metascutellum: apex protruding dorsally. Femoral depression: centrally smooth, peripherally foveolate. Leg color: coxae and at least the last three tarsomeres dark, otherwise reddish. Anterior corner of lateral propodeal carina: flat, without tooth. Posteromedial corner of lateral propodeal area: protruding posteriorly.

Metasoma color: T2 to flat part of T4, troughs of S1 to variable part of S5 reddish, otherwise dark. Median lobe of T1: with 7 or more longitudinal carinae. Metasoma at middle of T4: with metasomal bend and abrupt transition in sculpture. Posterolateral margins of metasomal terga: without protrusions. T5 median carina: absent. Longitudinal sculptural septa on T5: strong, sharply raised. Transverse sculptural septa on T5: weak or absent, much weaker than the longitudinal septa. T5 setae: not directed posteriorly, arising from center of sculptural mesh. T6: longer than broad. T6 laterotergite: overlapping S6. S4 median carina: present. Transverse sculptural septa on S5: weak or absent, much weaker than the longitudinal septa. S5 setae: not directed posteriorly, arising from center of sculptural mesh. Lateral carinae of S6: not meeting apically. Apex of S6: without notch.

*Male*. unknown.

##### Diagnosis.

*Female*. A6 with 2 large ventral sensilla; 3 mandibular teeth, with middle tooth much smaller than the others; metascutellum broadly trapezoidal with a broad truncate apex; metasomal bend present; sculpture posterior to metasomal bend strong longitudinal septa and weaker transverse septa; T4–T6 and S4–S6 with or without median carina; T6 longer than broad; S6 without apical notch. This species is very similar to *Bracalba magnirubra* in coloration and in having a relatively large body, but it differs in many characters, especially in lacking the apical S6 notch in females.

##### Etymology.

Used as a noun in apposition to the generic name, derived from a combination of the Latin participle sculptus and noun frons, referring to its strong network of frontal carinae.

##### Link to distribution map.

http://hol.osu.edu/map-full.html?id=302161

##### Material examined.

Holotype, female: AUSTRALIA: WA, ~25km E Perth, John Forrest National Park, 23.XII-27.XII.1986, J. S. Noyes, OSUC 231799 (deposited in WAMP). Paratype: AUSTRALIA: 1 female, OSUC 148704 (CNCI).

#### Bracalba sparsa

Burks sp. n.

urn:lsid:zoobank.org:act:C947CBC7-D26E-4001-AE11-3A8EDD4873A3

urn:lsid:biosci.ohio-state.edu:osuc_concepts:302162

http://species-id.net/wiki/Bracalba_sparsa

**Figures 55–59.**
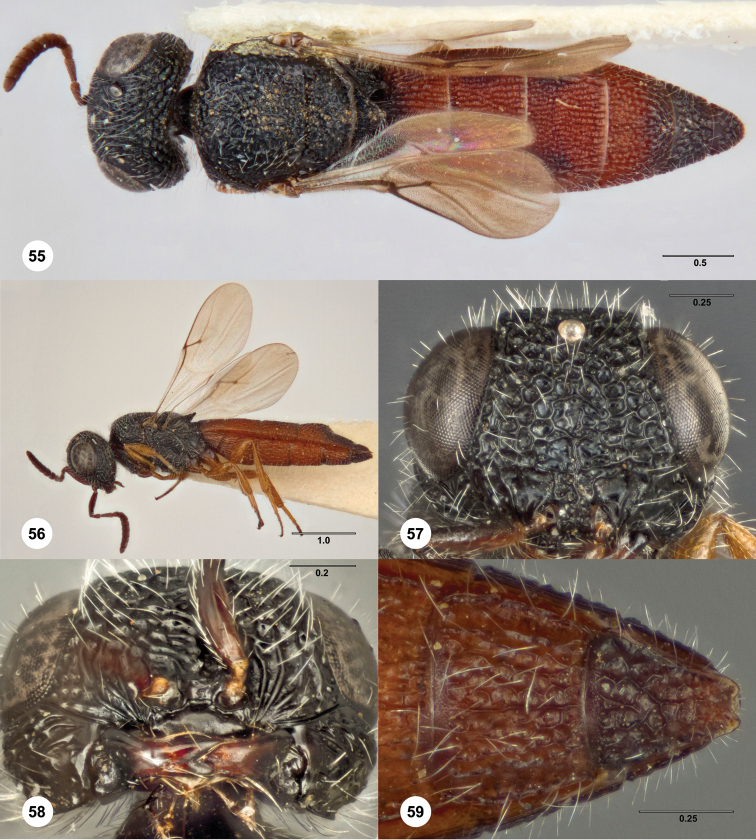
*Bracalba sparsa* sp. n.,holotype female (OSUC 231798). **55** Dorsal habitus **56** Lateral habitus **58** Head, ventral view, paratype female (OSUC 238205) **57** Head, anterior view **59** Metasomal sterna 5–6, ventral view. Morphbank^28^

Figures 55–59 Morphbank ^28^

##### Description.

*Female*. Body length 3.37–4.00 mm (n=6). Color of antenna beyond radicle: reddish-brown, darker at scape apex, pedicel, and A3. Radicle color: lighter than scape. Number of claval segments with ventral gustatory sensilla: 7. Number of ventral gustatory sensilla on A6: 1.

Ocular setae: long and sparse. Frontal depression: with irregular rugae indicating large foveae. Smooth depression extending dorsolaterally from antennal foramen: present. Dorsal clypeal margin: forming a complete connection between antennal foramina medially. Clypeal median carina: absent. Ventral clypeal margin: with a small median point. Mandibular color: dark basally and at teeth, becoming lighter reddish brown between these areas. Mandibular teeth: two, separated by narrow incision. Smooth area obliquely posterior to lateral ocellus: present. Genal sculpture: reticulate-rugose without any strong carinae.

Dorsal pronotal area: not set off by carina ventrally. Anterolateral corner of dorsal pronotal area: truncate anteriorly. Sculpture of posteromedian area of mesoscutum: densely foveolate. Lateral margin of dorsal axillar area: with a semicircular expansion, broadest near midlength. Mesoscutellar sculpture: densely foveolate. Metascutellum in dorsal view: trapezoidal but tapering to narrow apex. Dorsal surface of metascutellum: apex protruding dorsally. Femoral depression: crossed by rounded carinae. Leg color: entirely reddish. Anterior corner of lateral propodeal carina: flat, without tooth. Posteromedial corner of lateral propodeal area: protruding posteriorly.

Metasoma color: mostly reddish, variably dark at T4 bend, T5-T6, with variable smaller dark areas elsewhere. Median lobe of T1: with 7 or more longitudinal carinae. Metasoma at middle of T4: with metasomal bend and abrupt transition in sculpture. Posterolateral margins of metasomal terga: with tooth-like protrusions. T5 median carina: absent. Longitudinal sculptural septa on T5: strong, sharply raised. Transverse sculptural septa on T5: weak or absent, much weaker than the longitudinal septa. T5 setae: not directed posteriorly, arising from center of sculptural mesh. T6: as broad or broader than long. T6 laterotergite: overlapping S6. S4 median carina: present. Transverse sculptural septa on S5: weak or absent, much weaker than the longitudinal septa. S5 setae: not directed posteriorly, arising from center of sculptural mesh. Lateral carinae of S6: not meeting apically. Apex of S6: with notch.

*Male*. Body length 3.5 mm (n=1). Flagellomere length: A3 over 1.5× as long as broad, most others as long or longer than broad. T7: arched and posteriorly concave.

##### Diagnosis.

*Female*. A6 with 1 large ventral sensillum; 2 mandibular teeth; metascutellum subtrapezoidal but strongly tapering to a narrow truncate apex; metasomal bend expressed as a raised hump with posterior metasomal segments not tilted downwards; sculpture posterior to metasomal hump with strong longitudinal and transverse septa; T4–T6 without longitudinal carina; T6 broader than long; S4–S6 with longitudinal carina; S6 with apical notch.

##### Etymology.

Latin participle meaning “scattered,” recalling the sparsely distributed setae of this species.

##### Link to distribution map.

http://hol.osu.edu/map-full.html?id=302162

##### Material examined.

Holotype, female: AUSTRALIA: WA, Toodyay, 31.X.1979, R. M. Bohart, OSUC 231798 (deposited in WAMP). Paratypes: AUSTRALIA: 4 females, 3 males, OSUC 230821 (ANIC); OSUC 148703, 238204-238206, 238208 (CNCI); OSUC 55870 (OSUC).

##### Comments.

*Bracalba sparsa* represents a distinctive element within the genus, with its unique metasoma and unusual metascutellum.

#### Bracalba tricorata

Burks sp. n.

urn:lsid:zoobank.org:act:D54F676D-1EEF-4B40-88DA-CFB9583628C5

urn:lsid:biosci.ohio-state.edu:osuc_concepts:302163

http://species-id.net/wiki/Bracalba_tricorata

**Figures 60–61.**
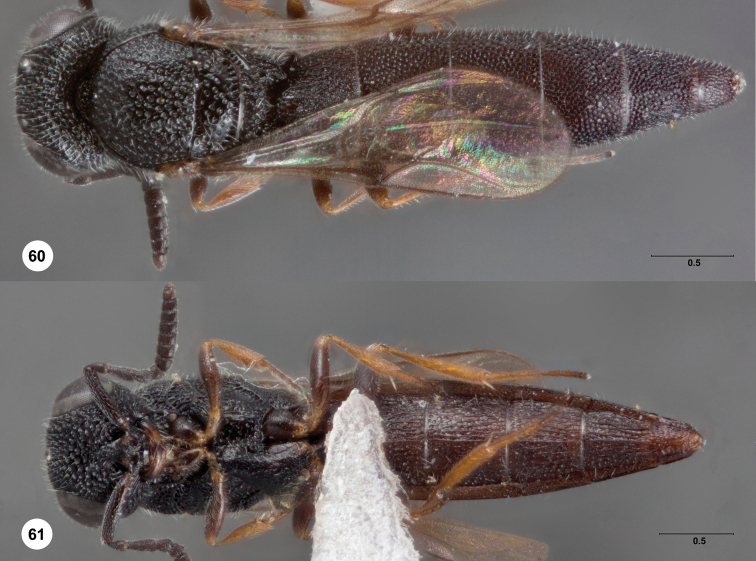
*Bracalba tricorata* sp. n.,holotype female (OSUC 238541). **60** Dorsal habitus **61 **Ventral habitus. Morphbank^29^

Figures 60–61 Morphbank ^29^

##### Description.

*Female*. Body length 3.88–4.25 mm (n=4). Color of antenna beyond radicle: entirely dark. Radicle color: same as scape. Number of claval segments with ventral gustatory sensilla: 7. Number of ventral gustatory sensilla on A6: 1.

Ocular setae: short and sparse. Frontal depression: with many strong transverse carinae, sparsely foveolate at torular triangle. Smooth depression extending dorsolaterally from antennal foramen: present. Dorsal clypeal margin: bordering antennal foramina, absent between them. Clypeal median carina: absent. Ventral clypeal margin: with a small median point. Mandibular color: mostly yellowish brown, dark at teeth. Mandibular teeth: three, but middle tooth tiny. Smooth area obliquely posterior to lateral ocellus: present. Genal sculpture: deeply reticulate-rugose with some septa much stronger than others, forming distinct rows differing in height.

Dorsal pronotal area: not set off by carina ventrally. Anterolateral corner of dorsal pronotal area: weakly rounded anteriorly. Sculpture of posteromedian area of mesoscutum: foveolate with slightly stronger longitudinal septa. Lateral margin of dorsal axillar area: with a semicircular expansion, broadest near midlength. Mesoscutellar sculpture: sparsely foveolate, with large smooth interspaces. Metascutellum in dorsal view: trapezoidal with broad apex. Dorsal surface of metascutellum: convex. Femoral depression: crossed by rounded carinae. Leg color: coxae, femora (aside from their apices), and at least the last two tarsomeres dark, otherwise yellowish brown. Anterior corner of lateral propodeal carina: flat, without tooth. Posteromedial corner of lateral propodeal area: protruding posteriorly.

Metasoma color: black to dark reddish brown. Median lobe of T1: with a set of rugae that merge with one another. Metasoma at middle of T4: with very weak bend. Posterolateral margins of metasomal terga: without protrusions. T5 median carina: absent. Longitudinal sculptural septa on T5: weak, blunt and hardly raised. Transverse sculptural septa on T5: about as strong as the longitudinal septa. T5 setae: directed posteriorly, arising from anterior edge of sculptural mesh. T6: as broad or broader than long. T6 laterotergite: overlapped by rim from S6. S4 median carina: absent. Transverse sculptural septa on S5: weak or absent, much weaker than the longitudinal septa. S5 setae: directed posteriorly, arising from anterior edge of sculptural mesh. Lateral carinae of S6: forming complete peripheral carina. Apex of S6: without notch.

*Male*. Body length 3.75–4.00 mm (n=3). Flagellomere length: A3 over 1.5× as long as broad, most others as long or longer than broad. T7: arched and posteriorly concave.

##### Diagnosis.

*Female*. A6 with 1 large ventral sensillum; 3 mandibular teeth with the middle tooth much smaller than the others; metascutellum broadly trapezoidal with a broad apex that is truncate or sometimes slightly incised; metasomal bend present but very weak; sculpture posterior to metasomal bend with transverse septa about as strong as the longitudinal septa; T4–T6 without median carina, S4–S6 with or without median carina; T6 about as broad as long; S5 with longitudinal septa much stronger than transverse septa; S6 without apical notch. This species is very similar to *Bracalba pinnula*, but has distinctly different ventral metasomal sculpture.

##### Etymology.

Latin participle meaning “tricky,” named for its strong similarity to some other species and its very subtle metasomal bend.

##### Link to distribution map.

http://hol.osu.edu/map-full.html?id=302163

##### Material examined.

Holotype, female: AUSTRALIA: WA, 20km N Denmark, 16.I.1987, J. S. Noyes, OSUC 238541 (deposited in WAMP). Paratypes: AUSTRALIA: 3 females, 3 males, OSUC 148699, 238192, 238195, 238197, 238199-238200 (CNCI).

#### Bracalba tridentata

Burks sp. n.

urn:lsid:zoobank.org:act:193C973C-CFBA-48E9-8F82-34589E44F32E

urn:lsid:biosci.ohio-state.edu:osuc_concepts:302164

http://species-id.net/wiki/Bracalba_tridentata

**Figures 62–65.**
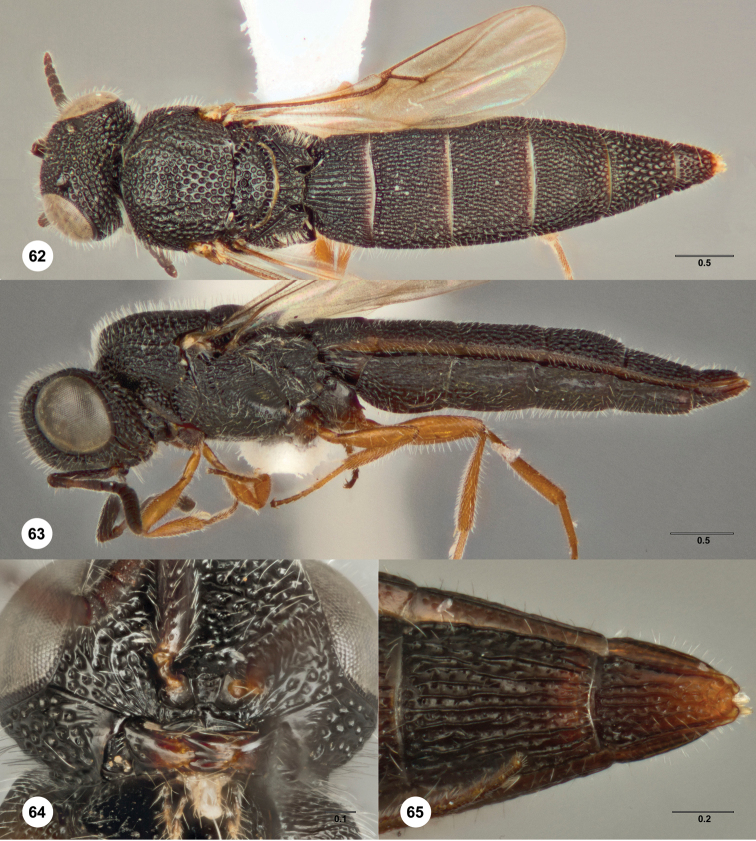
*Bracalba tridentata* sp. n.,paratype female (OSUC 238116). **62** Dorsal habitus **64 **Head, ventral view, metasomal sterna 5–6, ventral view **65** Metasomal sterna 5–6, ventral view, holotype female (OSUC 238113) **63** Lateral habitus. Morphbank^30^

Figures 62–65 Morphbank ^30^

##### Description.

*Female*. Body length 5.12–5.38 mm (n=3). Color of antenna beyond radicle: entirely dark. Radicle color: same as scape. Number of claval segments with ventral gustatory sensilla: 7. Number of ventral gustatory sensilla on A6: 1.

Ocular setae: long and sparse. Frontal depression: with many irregularly transverse rugae. Smooth depression extending dorsolaterally from antennal foramen: present. Dorsal clypeal margin: arched, interrupted by broad median carina. Clypeal median carina: present. Ventral clypeal margin: with a small median point. Mandibular color: dark basally and at teeth, becoming lighter reddish brown between these areas. Mandibular teeth: three, but middle tooth tiny. Smooth area obliquely posterior to lateral ocellus: present. Genal sculpture: deeply reticulate-rugose with some septa much stronger than others, forming distinct rows differing in height.

Dorsal pronotal area: not set off by carina ventrally. Anterolateral corner of dorsal pronotal area: truncate anteriorly. Sculpture of posteromedian area of mesoscutum: densely foveolate. Lateral margin of dorsal axillar area: with a semicircular expansion, broadest near midlength. Mesoscutellar sculpture: densely foveolate. Metascutellum in dorsal view: trapezoidal with broad apex, or elongate-trapezoidal but with incised apex. Dorsal surface of metascutellum: apex protruding dorsally. Femoral depression: crossed by 4-6 dorsal carinae, 7-8 ventral carinae interrupted by central smooth area. Leg color: yellowish-brown except for dorsal external part of coxae and sometimes tarsomeres 2-5. Anterior corner of lateral propodeal carina: flat, without tooth. Posteromedial corner of lateral propodeal area: protruding posteriorly.

Metasoma color: black to dark reddish brown. Median lobe of T1: with 7 or more longitudinal carinae. Metasoma at middle of T4: with metasomal bend and abrupt transition in sculpture. Posterolateral margins of metasomal terga: without protrusions. T5 median carina: absent. Longitudinal sculptural septa on T5: strong, sharply raised. Transverse sculptural septa on T5: about as strong as the longitudinal septa. T5 setae: not directed posteriorly, arising from center of sculptural mesh. T6: as broad or broader than long. T6 laterotergite: overlapping S6. S4 median carina: absent. Transverse sculptural septa on S5: weak or absent, much weaker than the longitudinal septa. S5 setae: not directed posteriorly, arising from center of sculptural mesh. Lateral carinae of S6: not meeting apically. Apex of S6: with notch.

*Male*. unknown.

##### Diagnosis.

*Female*. A6 with 1 large ventral sensillum; middle mandibular tooth present but much smaller than the others; metascutellum broadly trapezoidal and with a broadly truncate apex that may have a conspicuous median notch; metasomal bend strong; sculpture posterior to metasomal bend distinctly different from that anterior to it, with very weak transverse septa and strong longitudinal septa; T6 about as long as broad; S6 with apical notch. This species is very similar to *Bracalba hesperia*, but S6 in that species does not have an apical notch. It is also similar to *Bracalba sparsa*, but that species has a unique metasomal bend and metascutellar shape.

##### Etymology.

Latin adjective, named for the usually 3-pronged pattern formed apically by dark cuticle on T6.

##### Link to distribution map.

http://hol.osu.edu/map-full.html?id=302164

##### Material examined.

Holotype, female: AUSTRALIA: SA, 32km N Renmark, Amalia Dam, xeric mallee scrub, MT 4, ROM 2000041, Bookmark Biosphere Reserve, 33°53'S, 140°43'E, 263m, 15.II-15.IV.2000, malaise trap, D. C. Darling, OSUC 238113 (deposited in SAMA). Paratypes: AUSTRALIA: 2 females, OSUC 238114, 238116 (CNCI).

## Supplementary Material

XML Treatment for Bracalba

XML Treatment for Bracalba cuneata

XML Treatment for Bracalba globosa

XML Treatment for Bracalba plana

XML Treatment for Bracalba clavata

XML Treatment for Bracalba hesperia

XML Treatment for Bracalba intermedia

XML Treatment for Bracalba laminata

XML Treatment for Bracalba magnirubra

XML Treatment for Bracalba nigrescens

XML Treatment for Bracalba parvirubra

XML Treatment for Bracalba pinnula

XML Treatment for Bracalba propodealis

XML Treatment for Bracalba sculptifrons

XML Treatment for Bracalba sparsa

XML Treatment for Bracalba tricorata

XML Treatment for Bracalba tridentata

## Endnotes

1 urn:lsid:biocol.org:col:1008

2 urn:lsid:biocol.org:col:32975

3 urn:lsid:biocol.org:col:32981

4 urn:lsid:biocol.org:col:1009

5 urn:lsid:biocol.org:col:1012

6 urn:lsid:biocol.org:col:33928

7 urn:lsid:biocol.org:col:33791

8 urn:lsid:biocol.org:col:1014

9 urn:lsid:biocol.org:col:34157

10 urn:lsid:biocol.org:col:34161

11 urn:lsid:biocol.org:col:34982

12 urn:lsid:biocol.org:col:34411

13 urn:lsid:biocol.org:col:34578

14 urn:lsid:biocol.org:col:34593

15 http://www.morphbank.net/?id=799328

16 http://www.morphbank.net/?id=799329

17 http://www.morphbank.net/?id=799330

18 http://www.morphbank.net/?id=799331

19 http://www.morphbank.net/?id=799332

20 http://www.morphbank.net/?id=799333

21 http://www.morphbank.net/?id=799334

22 http://www.morphbank.net/?id=799335

23 http://www.morphbank.net/?id=799336

24 http://www.morphbank.net/?id=799337

25 http://www.morphbank.net/?id=799338

26 http://www.morphbank.net/?id=799339

27 http://www.morphbank.net/?id=799340

28 http://www.morphbank.net/?id=799341

29 http://www.morphbank.net/?id=799342

30 http://www.morphbank.net/?id=799343

31 http://lsid.tdwg.org/urn:lsid:biosci.ohio-state.edu:osuc_pubs:111

32 http://lsid.tdwg.org/urn:lsid:biosci.ohio-state.edu:osuc_pubs:20940

33 http://lsid.tdwg.org/urn:lsid:biosci.ohio-state.edu:osuc_pubs:21201

34 http://lsid.tdwg.org/urn:lsid:biosci.ohio-state.edu:osuc_pubs:131

35 http://lsid.tdwg.org/urn:lsid:biosci.ohio-state.edu:osuc_pubs:9560

36 http://lsid.tdwg.org/urn:lsid:biosci.ohio-state.edu:osuc_pubs:409

37 http://lsid.tdwg.org/urn:lsid:biosci.ohio-state.edu:osuc_pubs:404

38 http://lsid.tdwg.org/urn:lsid:biosci.ohio-state.edu:osuc_pubs:21506

39 http://lsid.tdwg.org/urn:lsid:biosci.ohio-state.edu:osuc_pubs:353

40 http://lsid.tdwg.org/urn:lsid:biosci.ohio-state.edu:osuc_pubs:463

41 http://dx.doi.org/10.1111/j.1096-0031.2008.00217.x

42 http://lsid.tdwg.org/urn:lsid:biosci.ohio-state.edu:osuc_pubs:8079

43 http://lsid.tdwg.org/urn:lsid:biosci.ohio-state.edu:osuc_pubs:633

44 http://lsid.tdwg.org/urn:lsid:biosci.ohio-state.edu:osuc_pubs:229

45 http://lsid.tdwg.org/urn:lsid:biosci.ohio-state.edu:osuc_pubs:657

46 http://lsid.tdwg.org/urn:lsid:biosci.ohio-state.edu:osuc_pubs:311

47 http://lsid.tdwg.org/urn:lsid:biosci.ohio-state.edu:osuc_pubs:21304

48 http://lsid.tdwg.org/urn:lsid:biosci.ohio-state.edu:osuc_pubs:21300

49 http://lsid.tdwg.org/urn:lsid:biosci.ohio-state.edu:osuc_pubs:240

50 http://lsid.tdwg.org/urn:lsid:biosci.ohio-state.edu:osuc_pubs:21213

51 http://lsid.tdwg.org/urn:lsid:biosci.ohio-state.edu:osuc_pubs:23583

52 http://lsid.tdwg.org/urn:lsid:biosci.ohio-state.edu:osuc_pubs:23221

53 http://lsid.tdwg.org/urn:lsid:biosci.ohio-state.edu:osuc_pubs:528

## Appendix 1

**Characters**

*Color of antenna beyond radicle*

1. entirely dark

2. entirely reddish

3. mostly dark, extreme base of scape becoming reddish

4. reddish-brown, darker at scape apex, pedicel, and A3

5. mostly dark, ventral parts of pedicel apex, A4-A12 variably lighter

6. yellowish-brown

7. dark except for pedicel apex

8. claval segments brown, basal segments yellowish

*Radicle color*

1. same as scape

2. base lighter than scape

3. lighter than scape

*Number of claval segments with ventral gustatory sensilla*

1. 6

2. 7

*Number of ventral gustatory sensilla on A6*

1. 1

2. 2

3. 0

*Ocular setae*

1. short and dense

2. long and sparse

3. short and sparse

4. long and dense

5. absent

*Frontal depression ventral sculpture*

1. with transverse rugae

2. with large areoles

3. smooth or foveolate

4. with median longitudinal carina

5. inapplicable (frontal depression absent)

*Frontal depression dorsal sculpture*

1. with transverse rugae

2. with large areoles

3. smooth or foveolate

4. with median longitudinal carina

5. inapplicable (frontal depression absent)

*Smooth depression extending dorsolaterally from antennal foramen*

1. absent

2. present

*Dorsal clypeal margin*

1. absent between antennal foramina

2. interrupted by a median areole below interantennal process

3. interrupted by median carina

4. complete only between antennal foramina medially

5. wrinkle-like with median peak

6. angular, emarginate medially

7. uniformly arched without interruption

*Clypeal median carina*

1. present

2. absent

*Ventral clypeal margin*

1. straight

2. with a small median point

3. convex

4. concave

*Mandibular color*

1. dark basally and at teeth, becoming lighter reddish brown between these areas

2. entirely dark

3. mostly yellowish brown, dark at teeth

4. mostly reddish brown, dark at teeth

*Mandibular teeth*

1. three of roughly equal size

2. two, separated by narrow incision

3. three, but middle tooth tiny

4. two, broadly separated by an angled flat area

*Smooth area obliquely posterior to lateral ocellus*

1. present

2. absent

*Genal sculpture*

1. reticulate-rugose with strong dorsoventral carinae

2. reticulate-rugose without any strong carinae

3. deeply reticulate-rugose with some septa much stronger than others, forming distinct rows differing in height

*Dorsal pronotal area*

1. not set off by carina

2. set off by carina

*Anterolateral corner of dorsal pronotal area*

1. protruding anteriorly

2. truncate anteriorly

3. weakly rounded anteriorly

*Sculpture of posteromedian area of mesoscutum*

1. sparsely foveolate, with large smooth interspaces

2. with many longitudinal carinae

3. densely foveolate

4. foveolate with broad longitudinal septa

5. foveolate with slightly stronger longitudinal septa

6. foveolate, with some interspersed additional tiny foveae

*Lateral margin of dorsal axillar area*

1. not expanded

2. broadly expanded

3. triangularly expanded or with posterior tooth, broadest posteriorly

4. with a semicircular expansion, broadest near midlength

*Mesoscutellar sculpture*

1. densely foveolate

2. sparsely foveolate, with large smooth interspaces

3. densely foveolate with one or two median longitudinal channels

4. densely foveolate but with smooth central area

5. densely foveolate with slightly stronger longitudinal septa

6. foveolate, with some interspersed additional tiny foveae

7. densely foveolate with a longitudinal carina

8. medially smooth except for a few irregular carinae

*Metascutellum in dorsal view*

1. strongly transverse, subrectangular

2. trapezoidal with broad apex

3. trapezoidal but tapering to narrow apex

4. elongate-trapezoidal but with incised apex

5. very short, subrectangular

6. semicircular

7. elongate-rectangular

*Dorsal surface of metascutellum*

1. flat

2. apex protruding dorsally

3. convex

*Femoral depression*

1. crossed by rounded carinae

2. irregularly foveolate but not crossed by carinae

3. smooth, not crossed by carinae

4. crossed by 4-6 dorsal carinae, 7-8 ventral carinae interrupted by central smooth area

5. centrally smooth, peripherally foveolate

6. shallowly sculptured but not crossed by carinae

7. crossed by many sharply defined carinae

*Leg color*

1. entirely dark

2. entirely reddish

3. coxae dark, all else reddish

4. coxae, femora (aside from their apices), and at least the last two tarsomeres dark, otherwise yellowish brown

5. coxae and at least the last three tarsomeres dark, otherwise reddish

6. reddish but with at least last two tarsomeres dark

7. dark except for 2nd trochanter of fore leg and mid leg, tip of mid and hind metatibia

8. reddish except for variable antero-dorsal portion of coxae

9. dark except for trochanters, tips of femora and tibiae, and tarsomeres 1-4

10. coxae and femora dark, but tibiae, tarsi, and sometimes 2nd trochanters yellowish brown

11. dark except for tips of tibiae and at least 1st tarsomere

12. yellowish-brown except for dorsal part of coxae and sometimes tarsomeres 2-5

13. dark except for tibiae, 2nd trochanter, and tarsomeres 2-5

14. dark, except trochanters, femoral apices, variable parts of tibiae, and tarsi pale

15. coxae, femora, and apical two tarsomeres dark, trochanters, tibiae, and other tarsomeres yellowish brown

16. coxae dark, leg becoming gradually lighter apically

17. coxae and at least the last three tarsomeres dark, otherwise yellowish

18. entirely yellowish

*Anterior corner of lateral propodeal carina*

1. flat, without tooth

2. with longitudinal tooth-like crest extending dorsally above level of metascutellum

*Posteromedial corner of lateral propodeal area*

1. not protruding posteriorly

2. protruding posteriorly

*Metasoma color*

1. black to dark reddish brown

2. entirely reddish

3. reddish, with last segment variably dark

4. reddish, with last two segments dark

5. reddish, with last 2.5 segments dark

6. T2 to flat part of T4, troughs of S1 to variable part of S5 reddish; otherwise dark

7. mostly reddish, variably dark at T4 bend, T5-T6, with variable smaller dark areas elsewhere

8. centrally reddish with some vague dark intrusions, peripherally becoming black

*Median lobe of T1*

1. with 7 or more longitudinal carinae

2. with a set of rugae that merge with one another

3. with median smooth area interrupting carinae

4. with 6 carinae

5. with 5 carinae

*Metasoma at middle of T4*

1. with metasomal bend and abrupt transition in sculpture

2. without bend

3. with very weak bend

*Posterolateral margins of metasomal terga*

1. with tooth-like protrusions

2. without protrusions

*T5 median carina*

1. absent

2. present

*Longitudinal sculptural septa on T5*

1. strong, sharply raised

2. weak, blunt and hardly raised

*Transverse sculptural septa on T5*

1. about as strong as the longitudinal septa

2. weak or absent, much weaker than the longitudinal septa

*T5 setae*

1. directed posteriorly, arising from anterior edge of sculptural mesh

2. not directed posteriorly, arising from center of sculptural mesh

*T6*

1. longer than broad

2. as broad or broader than long

*T6 laterotergite*

1. overlapped by rim from S6

2. overlapping S6

*S4 median carina*

1. absent

2. present

*Transverse sculptural septa on S5*

1. weak or absent, much weaker than the longitudinal septa

2. about as strong as the longitudinal septa

*S5 setae*

1. directed posteriorly, arising from anterior edge of sculptural mesh

2. not directed posteriorly, arising from center of sculptural mesh

*Lateral carinae of S6*

1. forming complete peripheral carina

2. not meeting apically

3. absent

4. weak and ruga-like, becoming indistinct posteriorly

*Apex of S6*

1. without notch

2. with notch

3. roundly concave

*Flagellomere length*

1. A3 over 1.5× as long as broad, most others as long or longer than broad

2. A3 1–1.2× as long as broad, most others transverse

*T7*

1. arched and posteriorly concave

2. flat and posteriorly truncate

### Matrix

Chromoteleia sp. 7002444010221101223052200H02111000100020011

Bracalba_cuneata 50110[23][23]16111000[01]132[04]11001B0[02]101001100020011

Bracalba_globosa 02110[23][23]161110100242111001C01101001100020011

Bracalba_plana 4211000101110200222150401F00101001001020111

Bracalba_clavata 41020[03][02]13112020023230[02]400300200001110020001

Bracalba_hesperia 00102021401001002232124018000[01]1000000120?1?

Bracalba_intermedia 0202022061220100242400401301101000110020011

Bracalba_laminata ??11302101120200143631001???????????????0?0

Bracalba_magnirubra 221012211012010002303[12]001520010110110111000

Bracalba_nigrescens ????300101120230203131001G??????????????0?0

Bracalba_parvirubra 201012012012020004323[02]601B70000001010000?0?

Bracalba_pinnula 021[01]00010110020022301[02]401901001001111020011

Bracalba_sculptifrons 00110[13][13]150120000123011401450000110110110?1?

Bracalba_propodealis 0110022101120200233142111300001000111120?1?

Bracalba_tricorata 0010200101120220243112001301201001000000010

Bracalba_sparsa 3210122131110100123021001160000111110111000

Bracalba_tridentata 00101001201202001230[13]1301B00000011100111?1?

## Appendix 2

Taxonomic records for all records used in the present paper. (doi: 10.3897/zookeys.236.3434.app2) File format: DarwinCore Archive.

**Explanation note:** Darwin Core Archive (DwC-A) is the preferred format for publishing data to the Global Biodiversity Information Facility (GBIF) network. Together with Dublin Core (on which its ideas are based), it is used by GBIF and others to encode data about organism names, taxonomies and species information.

## Appendix 3

Locality records for all records used in the present paper. (doi: 10.3897/zookeys.236.3434.app3) File format: DarwinCore Archive.

**Explanation note:** Darwin Core Archive (DwC-A) is the preferred format for publishing data to the Global Biodiversity Information Facility (GBIF) network. Together with Dublin Core (on which its ideas are based), it is used by GBIF and others to encode data about organism names, taxonomies and species information.
